# Characterization of velvet family proteins regulating fungal development and virulence in the peach shoot blight fungus *Diaporthe amygdali*

**DOI:** 10.1080/21505594.2026.2715219

**Published:** 2026-08-14

**Authors:** Hengsong Shi, Jingpu Hao, Xun Liu, Yue Zhang, Lina Yang, Zhaolin Ji

**Affiliations:** College of Plant Protection, Yangzhou University, Yangzhou, Jiangsu, China

**Keywords:** *Diaporthe amygdali*, velvet protein, development, pathogenicity, secondary metabolism

## Abstract

Peach shoot blight, caused by *Diaporthe amygdali*, is a serious challenge for China’s peach industry, contributing to annual yield losses of 20–50%. Despite its economic significance, the molecular mechanisms underlying *D. amygdali* pathogenicity remain poorly characterized. Velvet proteins are fungal-specific regulatory factors that exhibit functional diversity across plant pathogenic fungi; however, their roles in *D*. *amygdali* remain uncharacterized. Here, we identified and systematically characterized four velvet proteins in *D*. *amygdali*, including DaVeA, DaVelB, DaVelC, and DaVosA. Expression analysis revealed that *DaVEA*, *DaVELB*, and *DaVELC* exhibited significantly higher expression during the asexual stage; notably, *DaVEA* and *DaVELB* maintained markedly upregulated expression at the invasive stages. We further obtained single-gene velvet protein deletion mutants and their complemented strains, finding that deletion of *DaVEA* or *DaVELB* significantly reduced vegetative growth, abolished asexual reproduction, weakened the pathogenicity, increased melanin accumulation, altered colony surface hydrophobicity, and compromised stress tolerance. In contrast, deletion of *DaVELC* or *DaVOSA* affected only asexual reproduction. We further observed that deletion of *DaVEA* or *DaVELB* led to significant downregulation of the genes encoding cell wall-degrading enzymes and the fusicoccin biosynthetic gene cluster–a phytotoxic diterpene metabolite implicated in fungal virulence. Moreover, the four velvet proteins form complex interactions in *D. amygdali*. Our findings expand the functional repertoire of velvet proteins in fungal phytopathogens and clarify DaVeA and DaVelB as potential molecular targets for future studies aimed at developing control strategies.

## Introduction

Peach (*Prunus persica*) has been cultivated for over 4, 000 years and remains one of China’s most beloved and economically vital fruit trees [1]. Since 2011, peach shoot blight (PSB) has gradually appeared across multiple provinces in China, posing a growing challenge for growers, annual yield losses have ranged from 20–50% [2]. Early signs are brown, slightly sunken lesions that often appear at the base of new shoots. As the disease progresses, these lesions extend upward along the shoot, accompanied by yellowing leaves, wilting, and eventual leaf drop. In advanced cases, affected shoots dry out, become brittle, and might break away; severely impacting the whole tree. PSB may also affect buds and developing fruits, further impacting orchard health and harvest quality. Beyond China, PSB has been documented in several countries, including France, Italy, Tunisia, Greece, Portugal, Spain, Japan, the United States, and South Africa [3–8]. While several fungi, including *Diaporthe amygdali*, *Diaporthe eres*, *Phomopsis liquidambaris*, *Botryosphaeria obtusa*, and *Cytospora* spp., have been linked to PSB-like symptoms [9–11], *D. amygdali* stands out as the most consistently identified and predominant cause of PSB in China [12,13]. Notably, this pathogen also affects other stone fruits within the *Rosaceae* family, such as almond, pear, plum, and apricot, making its management especially important for regional fruit production [14–16]. However, the molecular mechanisms governing *D. amygdali* pathogenicity remain uncharacterized.

Velvet proteins are fungal-specific regulators that orchestrate fungal development and secondary metabolism. They typically include four well-conserved members: VeA, VelB, VelC, and VosA. Among these, VeA was first discovered in *Aspergillus nidulans*, and named for the soft, velvet-like appearance of its mycelium in deletion mutants. In *A. nidulans*, VeA plays a thoughtful balancing role: it gently encourages sexual reproduction while naturally tempering asexual development [17,18]. Similar to *A. nidulans*, its homolog in *Neurospora crassa* also acts as negative regulator of conidiation [19]. In plant pathogenic fungi, including *Botrytis cinerea*, *Fusarium verticillioides* and *Cochliobolus heterostrophus*, VeA homologs have also been proved to negatively regulate asexual development [20–23]. However, in *Aspergillus parasiticus* and *Fusarium fujikuroi*, VeA positively regulates conidial production and sclerotia formation [24,25]. Moreover, VeA also regulates the production of secondary metabolites in various fungi. In *Valsa mali*, *B. cinerea*, *Cochliobolus sativus* and *Mycosphaerella graminicola*, VeA and VelB negatively regulate melanin synthesis [23,26–28]. Besides that, in *A. nidulans*, *A. flavus* and *A. parasiticus*, VeA plays a key role in regulating aflatoxin production [24,29,30]. Additionally, VeA has been demonstrated to be a positive regulator of penicillin and PR-toxin in *Penicillium chrysogenum* [31]. In *Fusarium graminearum*, VeA is also proved to be capable of affecting the production of deoxynivalenol (DON) by regulating the expression of key genes in the biosynthesis pathway [32,33]. In addition, VeA interacts with VelB to move into the nucleus in the dark. Once there, they interact with the non-velvet protein LaeA to form a heterotrimeric complex, and co-regulate fungal development and secondary metabolites by adjusting chromatin accessibility and transcriptional activity [34,35]. Besides that, VelB could also form VelB/VosA homodimeric or heterodimeric complexes that negatively regulate conidiation in *A. nidulans, C. heterostrophus*, and *C. sativus* [36–38]. Moreover, VelB negatively regulates the conidial production in *V. mali*, *Fusarium oxysporum,* and *F. graminearum* [28,39,40]. However, in *Magnaporthe oryzae* and *Aspergillus ochraceus*, VelB positively regulates the conidiation, whereas MoVosA appears dispensable for fungal development [41,42]. VelC has been characterized in only a limited number of fungal species. In *M. oryzae*, knocking out *MoVELC* significantly reduces the conidia production and size [42]. In *A. nidulans*, deleting *VELC* means fewer sexual fruiting bodies form, and enhances the expression of genes associated with conidia production [43]. And in *P. chrysogenum*, deletion of *PcVELC* boosts conidiation [44].

More importantly, multiple studies have demonstrated that velvet proteins are essential regulators of virulence in plant-pathogenic fungi. In *M. oryzae*, MoVeA and MoVelC affect the pathogenicity by impairing appressorium function [42]. In *F. graminearum* and *V. mali*, deletion of *VEA* or *VELB* significantly attenuates virulence [28,33,39]. Similarly, velvet proteins have been shown to contribute to virulence in *F. oxysporum* [40], *Ustilago maydis* [45] and *Histoplasma capsulatum* [46]. Nevertheless, the functional roles of velvet proteins in *D. amygdali* remain uncharacterized.

In this study, we generated four single-gene velvet protein deletion mutants in *D. amygdali* and systematically characterized their individual and overlapping roles. Our results revealed that DaVeA and DaVelB are required for vegetative growth, asexual reproduction, virulence, melanin biosynthesis, response to multiple abiotic stresses, and expression of genes in the surface hydrophobicity, cell-wall degrading enzymes, and fusicoccin biosynthetic gene cluster. In contrast, DaVelC and DaVosA exhibit more restricted functions, contributing exclusively to asexual reproduction.

## Materials and methods

### Strains and culture conditions

ZN32 strain, isolated from the diseased peach shoots exhibiting typical symptoms of shoot blight in Jiaxing City, Zhejiang Province, China, was used as the wild-type control. All strains were inoculated on potato dextrose agar (PDA), complete medium (CM), oatmeal medium (OM), and alfalfa decoction-czapek medium (AC) plates at 25°C in the dark. TB3 media with 300 μg/mL hygromycin B or 200 μg/mL zeocin were used to select transformants. Peach variety “Liu Tiao Bai Feng” was used for pathogen inoculation. Peach and apple fruits were purchased from the fruit stores.

### Construction of replacement fragments and Cas9-gRNA vectors

Gene replacement constructs were generated using the split-PCR approach, comprising a complete hygromycin B resistance cassette flanked by approximately 1000 bp of upstream and downstream homologous sequences of the target gene [47]. For CRISPR-Cas9 vector construction, target-specific spacers were designed using the online CRISPR design tool (http://grna.ctegd.uga.edu/). Complementary sense and antisense oligonucleotides were then annealed and cloned into the *BsmB*I-v2–linearized pCas9-tRp-gRNA vector. All primers used in this study are listed in Supplementary Table S1.

### Generation of gene knock out and complemented strains

Polyethylene glycol (PEG)-mediated protoplast transformation was employed to generate gene knock out and complemented strains, following a previously established protocol [48]. Mycelial pellets cultured in liquid CM medium for 36 h were enzymatically lysed with lysing enzymes for 1.5 h to lysis cell wall. Protoplasts were pelleted by centrifugation, and resuspended in sorbitol–Tris–CaCl^2^ (STC) buffer. Subsequently, 25 μL of each of two overlapping PCR products and 12 μL of the Cas9-gRNA vector were co-transformed into 150 μL of ZN32 protoplasts and co-incubated for 25 min at room temperature. Later, 1 mL of PTC solution (60% w/v PEG4000 in STC buffer) was added to the protoplasts and incubated for an additional 25 min. Next, 8 mL of the liquid TB3 medium was added, and the mixture was incubated for 2 h at 25°C with gentle shaking at 70 rpm. Transformants were selected on solid TB3 medium supplemented with hygromycin B to isolate gene knockout strains.

For complementation, the full-length sequence of the target gene fused with the green fluorescent protein (GFP) encoding sequence was cloned into *Xho*I-digested pYF11 vector under the control of RP27 promoter using yeast gap repair approach. The complemented plasmid (12 μL) was then transferred into the corresponding gene knock out strains using PEG-mediated protoplast transformation. Transformants were selected on solid TB3 medium containing of zeocin-resistance.

### Vegetative growth and stress response assay

The wild-type strain ZN32, single-gene velvet protein deletion mutants (Δ*DaveA*, Δ*DavelB*, Δ*DavelC*, and Δ*DavosA*), and their corresponding complemented strains were re-actived for 3 days at 25°C in the dark. Hyphal blocks (2 mm × 2 mm) were cut from the colony margins of each strain and centrally inoculated onto PDA, CM, OM, and AC plates. All plates were incubated at 25°C in the dark for 5 days.

For stress response assay, hyphal blocks were inoculated onto CM plates supplemented with either 0.4 M NaCl, 0.6 M KCl, 1.0 M sorbitol, 400 µg/mL calcofluor white (CFW), 400 µg/mL Congo Red, 2.5 mM, or 5.0 mM H_2_O_2_. Non-supplemented CM plates served as controls for each strain. All plates were incubated at 25°C in the dark for 5 days.

### Virulence assay

Two-year-old peach twigs were cut into 7-cm segments, surface-sterilized with 75% (v/v) ethanol for 30 s, rinsed three times with sterile distilled water, and air-dried under aseptic conditions. Three equidistant, shallow wounds (3 mm deep) were made on each twig using a sterile syringe needle, and a 9-mm-diameter hyphal block of each tested strain was placed onto the wounds. The inoculated twigs were incubated in plastic boxes at 28°C under a 12-h light/12-h dark photoperiod and 90% relative humidity (RH) for 7 days.

For fruit inoculation, peach and apple fruits were treated following the same standardized protocol, including surface disinfection, wound creation, fungal inoculation, and incubation conditions. However, lesion development was assessed at 5 days post inoculation (dpi) on the peach fruits and at 20 dpi on apple fruits.

### DNA extraction and fungal biomass analysis

For fungal biomass quantification in infected peach and apple tissues, samples were collected from equivalent lesion areas inoculated with the wild-type strain ZN32, the knockout strains, or the corresponding complemented strains; each sample comprised both necrotic tissue and a 5-mm margin of adjacent healthy tissue. Samples were immediately frozen in liquid nitrogen and ground into a fine powder under liquid nitrogen. Total genomic DNA was extracted using the CTAB method. Fungal biomass was determined by DNA-based qRT-PCR targeting the *D. amygdali* histone H3-encoding gene *DaH3*, normalized within peach or apple translation elongation factor 2 gene *TEF2*. The fungal relative expression levels were calculated using the comparative Ct (2^−ΔΔCt^) method. Primers are listed in Supplementary Table S1.

### RNA extraction and gene expression analysis

Hyphal blocks (2 mm × 2 mm) of wild type ZN32 and single-gene velvet protein deletion mutants were cultured in liquid CM medium at 25°C for 36 h, respectively. Mycelia were harvested by filtration through a single-layer sterile gauze, briefly pressed to remove excess liquid, immediately flash-frozen in liquid nitrogen, and ground into a fine powder under liquid nitrogen. Total RNA was extracted using the PureLink RNA Mini Kit (Invitrogen, USA). First-strand cDNA was synthesized from 1 µg of DNase-treated total RNA using the HiScript II 1st Strand cDNA Synthesis Kit (R223-01; Vazyme, China). Transcript levels of genes involved in melanin biosynthesis, cell wall degradation, surface hydrophobicity, and fusicoccin biosynthesis were quantified by RNA-based qRT-PCR. The *DaH3* served as the endogenous reference gene. Primers are listed in Supplementary Table S1.

### Surface hydrophobicity assay

All tested strains were cultured on PDA plates for 5 days at 25°C. A 10-µL droplet of either detergent solution [contains 0.02% (w/v) SDS and 5 mM EDTA] or sterile distilled water was carefully placed onto the center of each colony. After 3 min of incubation at room temperature, droplet collapse was visually assessed.

### Yeast two-hybrid assay

For yeast two-hybrid (Y2H) assays, the open reading frame sequences of *DaVEA*, *DaVELB*, *DaVELC*, *DaVOSA*, and *DaLAEA* were amplified by PCR using cDNA of wild type strain ZN32 as the templated, respectively. Amplified fragments were cloned into *Nde*I- and *EcoR*I-linearized pGBKT7 (bait) or pGADT7 (prey) vectors via homologous recombination. Bait and prey plasmid pairs were co-transformed into *Saccharomyces cerevisiae* strain AH109 according to the manufacturer’s instructions. Transformants were first selected on synthetic dropout (SD) medium lacking leucine (Leu) and tryptophan (Trp) at 30°C for 3 days. Then, they were transferred into SD medium lacking histidine, leucine, tryptophan, and adenine for another 3 days. The pGBKT7-53/pGADT7-T pair used as the positive control, and the pGBKT7-Lam/pGADT7-T pair used as the negative control.

## Results

### Identification and phylogenetic analysis of velvet proteins in *D. amygdali*

**Figure 1.**
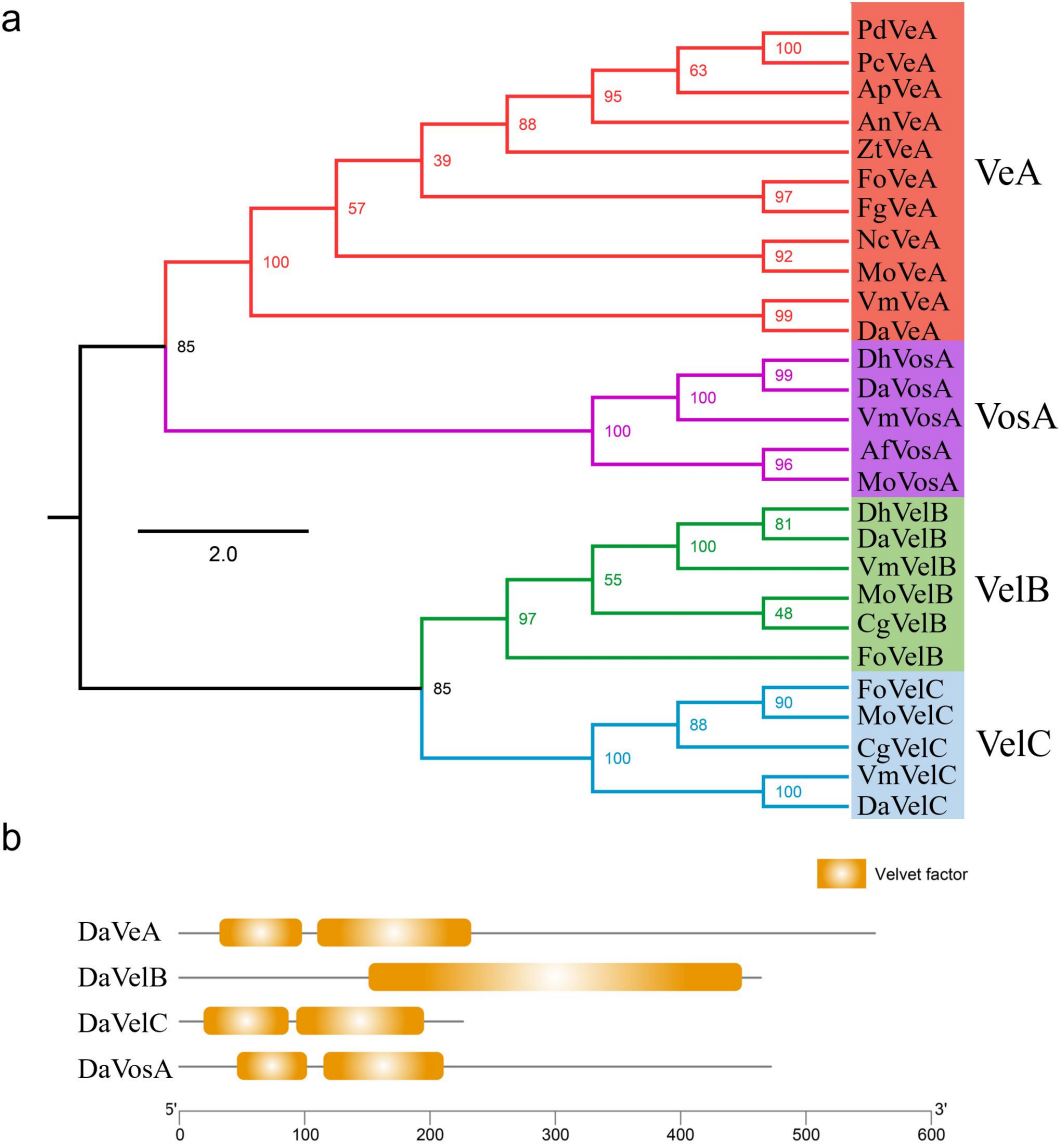
Phylogenetic analysis of velvet proteins and domain prediction. (a) A maximum-likelihood phylogenetic tree was constructed using the neighbor-joining method by MEGA11. Pd: *Penicillium digitatum*; Pc: *Penicillium citrinum*; Ap: *Aspergillus parasiticus*; An: *Aspergillus nidulans*; Zt: *Zymoseptoria tritici*; Fo: *Fusarium oxysporum*; fg: *Fusarium graminearum*; Nc: *Neurospora crassa*; Mo: *Magnaporthe oryzae*; Vm: *Valsa Mali*; Da: *Diaporthe amygdali*. (b) Domain of velvet proteins in *D. amygdali* were predicted using the conserved domain database (CDD) v3.20 via NCBI’s batch CD-Search tool.

Previous studies have shown that velvet proteins contribute to development and virulence in diverse plant-pathogenic fungi. Here, we identified velvet protein homologs of *V. mali* in *D*. *amygdali* using BLASTp against the *D. amygdali* genome. Four orthologous genes were identified, and designated as *DaVEA* (GME13000_g), *DaVELB* (GME1893_g), *DaVELC* (GME6461_g) and *DaVOSA* (GME3873_g). A maximum-likelihood phylogenetic tree was constructed using full-length amino acid sequences of velvet proteins from representative ascomycete species with MEGA11. Results revealed that strong evolutionary conservation among velvet proteins across fungal lineages and showed that the velvet protein homologs from *V. mali* are phylogenetically most closely related to those of *D. amygdali* (Figure 1(a)). Domain architecture analysis predicted that DaVeA, DaVelC, and DaVosA each contain two canonical velvet domains, whereas DaVelB harbors a single velvet domain (Figure 1(b)).

### Transcript analysis of velvet proteins at different stages of *D. amygdali*

**Figure 2.**
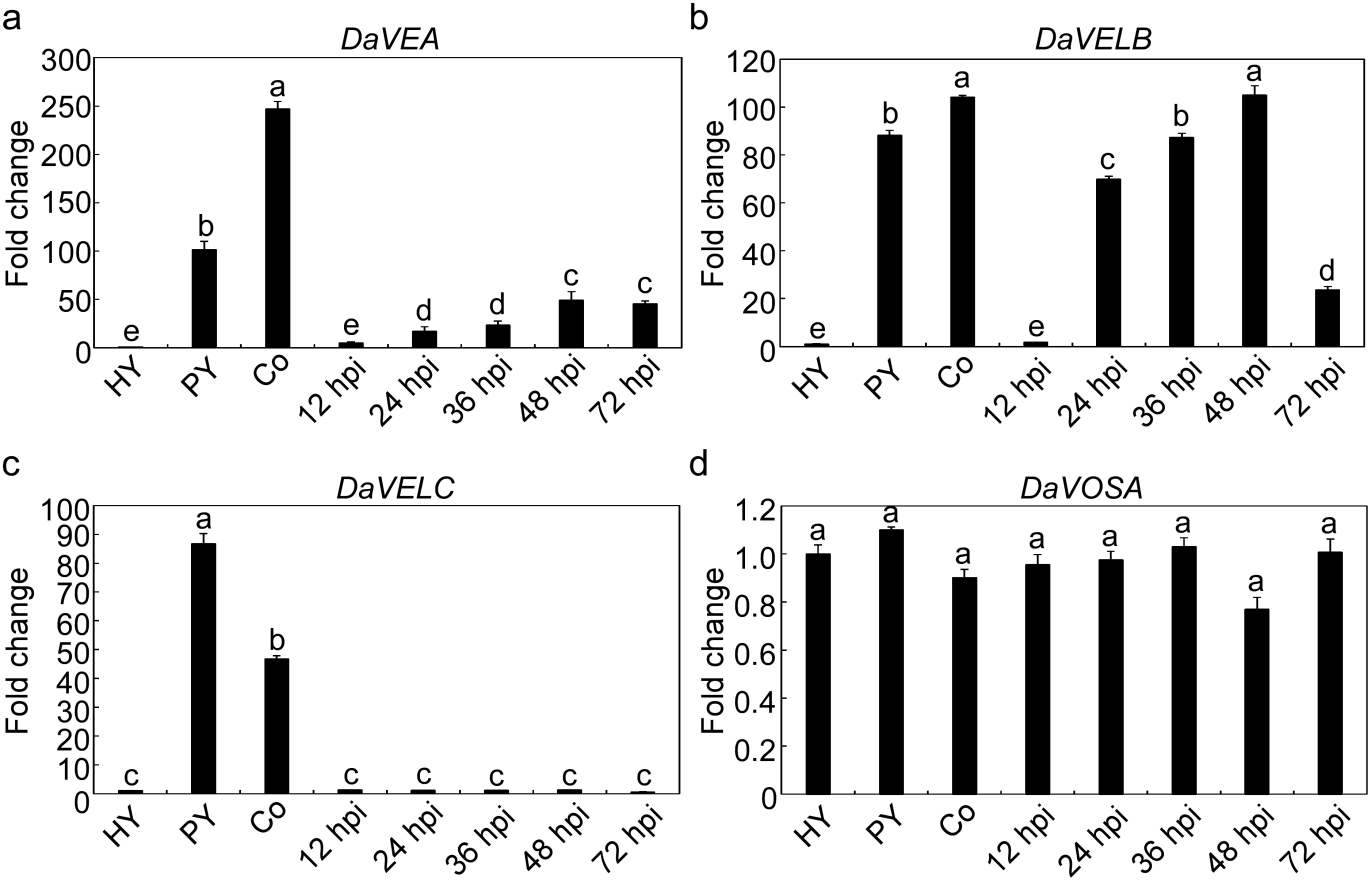
Transcriptional profiling of velvet protein-encoding genes across developmental and infected stages in *D. amygdali*. (a-d) Expression levels of velvet genes, including *DaVEA, DaVELB, DaVELC*, and *DaVOSA*, were quantified by qRT-PCR in vegetative hyphae (HY), pycnidia (PY), conidia (co), and infected peach twigs at 12, 24, 36, 48, and 72 hours post-inoculation (hpi). The experiments were repeated three times with the same results. Error bars represent standard deviations of data from three replicates. Normality and homogeneity of variance were checked before ANOVA; no data transformation was required. Lowercase indicates statistically significant differences (Student–Newman–Keuls test, *p* < 0.05).

We first examined the transcriptional profiles of velvet protein-encoding genes across developmental and infection stages, including vegetative hyphae (HY), pycnidia (PY), conidia (Co), and infected peach twigs at 12, 24, 36, 48, and 72 hours post-inoculation (hpi). Quantitative RT-PCR showed that, relative to hyphae, *DaVEA*, *DaVELB*, and *DaVELC* were significantly upregulated by at least 50-fold during pycnidial and conidial stages (Figure 2(a–c)). During infection, transcript levels of *DaVEA* and *DaVELB* increased progressively from 24 to 72 hpi; and the expression of *DaVELB* was much stronger than that of *DaVEA* (Figure 2(a,b)). In contrast, although *DaVELC* expression was strongly elevated in PY and Co stages, it remained unchanged throughout infection stages relative to the hyphal stages (Figure 2(c)). The expression of *DaVOSA* exhibited no significant difference across all stages (Figure 2(d)). Results indicate that DaVeA, DaVelB, and DaVelC might contribute to asexual reproduction, while DaVeA and DaVelB additionally play roles in the pathogenicity of *D. amygdali*.

### DaVeA and DaVelB regulate the vegetative growth of *D. amygdali*

**Figure 3.**
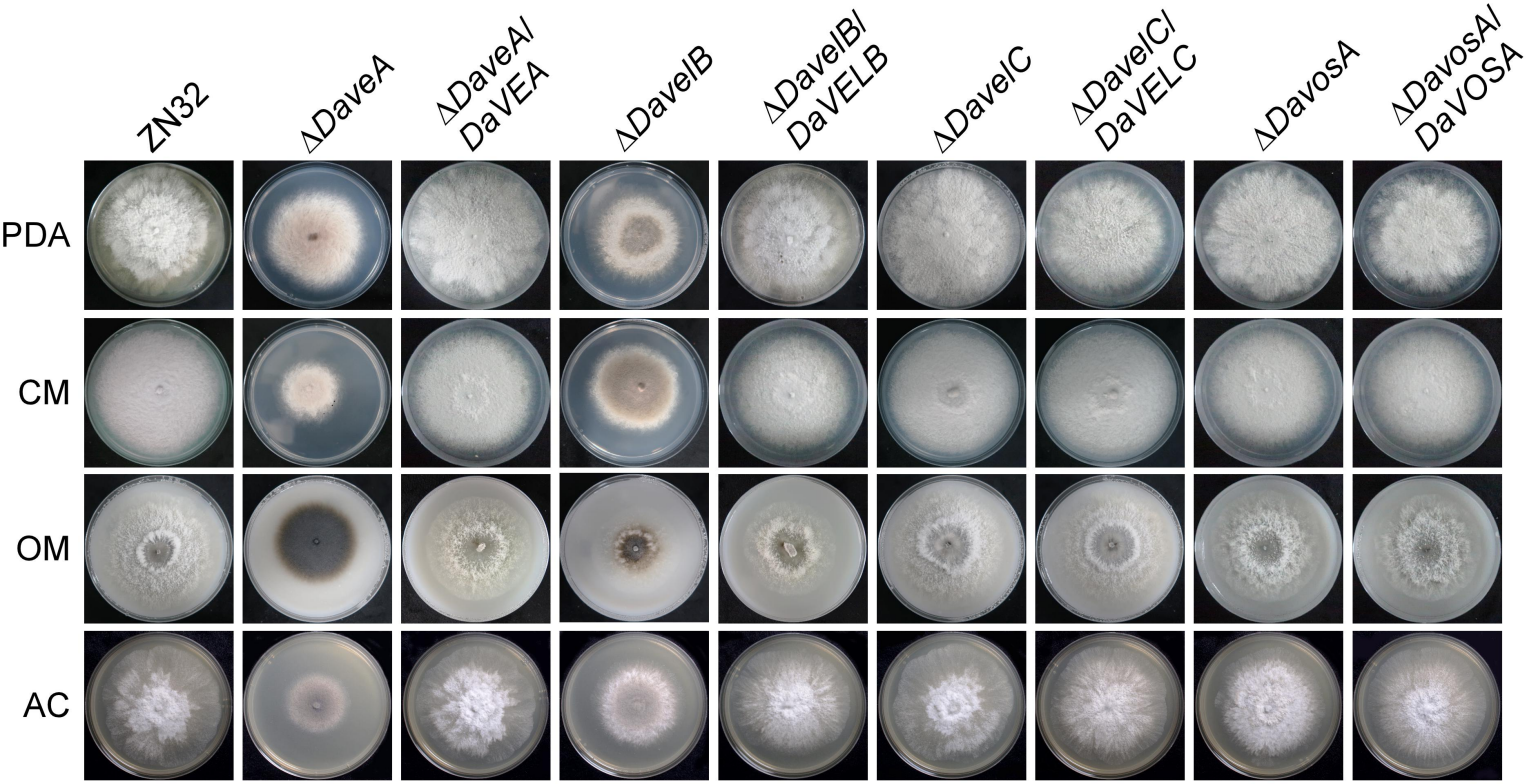
DaVeA and DaVelB regulate vegetative growth in *D. amygdali*. Wild-type, single-gene velvet protein deletion mutants, and their complemented strains were cultured on potato dextrose agar (PDA), complete medium (CM), oatmeal agar (OM), and alfalfa decoction–Czapek medium (AC) at 25°C under a 12-h light/12-h dark photoperiod for 5 days, respectively. The experiments were repeated three times with the same results.

To investigate the roles of velvet proteins in *D. amygdali*, single-gene deletion mutants (Δ*DaveA*, Δ*DavelB*, Δ*DavelC*, and Δ*DavosA*) were generated via PEG-mediated protoplast transformation by replacing each target gene with a hygromycin B phosphotransferase (hph) cassette (Figure S1). The complemented strains were subsequently obtained. We first assessed the contribution of velvet proteins to vegetative growth. The wild-type strain ZN32, single-gene velvet protein deletion mutants, along with their corresponding complemented strains, were cultured on PDA, CM, OM, and AC medium at 25°C in the dark, respectively. After 5 days, ZN32, Δ*DavelC*, Δ*DavosA* mutants, and the complemented strains presented irregular, fluffy, white aerial hyphae in the middle on the four kinds of plates, however, the colonies of Δ*DaveA* and Δ*DavelB* were dense within increased pigment in the middle, particularly on OM plates (Figure 3). In addition, the radial growth rates of Δ*DaveA* and Δ*DavelB* mutants exhibited significantly reduced compared to ZN32 and their complemented strains on all four kinds of plates. In contrast, no significant differences in growth rate were observed among Δ*DavelC* and Δ*DavosA* mutants (Table 1). These results illustrate that DaVeA and DaVelB are essential for vegetative growth of *D. amygdali*, whereas DaVelC and DaVosA are dispensable for this process.

**Table 1.** Phenotypic measurements of *D. amygdali* single-gene velvet protein deletion mutants.

Strain	Colony diameter (cm)				Number of pycnidia	
	PDA	CM	OM	AC	15 d	30 d
ZN32	8.1 ± 0.1a	8.1 ± 0.1a	6.2 ± 0.1a	6.4 ± 0.1a	130 ± 1.5a	137 ± 7.6a
Δ*DaveA*	5.1 ± 0.3c	4.8 ± 0.1c	4.5 ± 0.2b	3.2 ± 0.1c	0c	0c
Δ*DaveA*/*DaVEA*	8.1 ± 0.1a	8.1 ± 0.2a	6.1 ± 0.1a	6.4 ± 0.1a	128 ± 4.0a	131 ± 11.0a
Δ*DavelB*	6.1 ± 0.2b	6.4 ± 0.1b	4.0 ± 0.1c	4.9 ± 0.1b	0c	0c
Δ*DavelB*/*DaVELB*	8.1 ± 0.1a	8.0 ± 0.1a	6.1 ± 0.1a	6.2 ± 0.2a	128 ± 3.1a	133 ± 5.7a
Δ*DavelC*	8.0 ± 0.1a	8.1 ± 0.1a	6.1 ± 0.1a	6.4 ± 0.1a	61 ± 2.1b	57 ± 7.1b
Δ*DavelC*/*DaVELC*	7.7 ± 0.1a	8.1 ± 0.1a	6.1 ± 0.1a	6.5 ± 0.1a	132 ± 2.1a	136 ± 8.6a
Δ*DavosA*	8.0 ± 0.1a	8.1 ± 0.1a	6.1 ± 0.2a	6.4 ± 0.1a	133 ± 5.5a	129 ± 11.0a
Δ*DavosA*/*DaVOSA*	8.0 ± 0.1a	8.0 ± 0.2a	6.2 ± 0.1a	6.5 ± 0.1a	130 ± 4.0a	133 ± 4.0a

Colony diameters of all test strains were measured on potato dextrose agar (PDA), complete medium (CM), oatmeal agar (OM), and alfalfa decoction–czapek agar (AC) at 25°C under a 12-h light/12-h dark photoperiod at 5 days post-inoculation. The number of pycnidia of tested strains was measured at 15 and 30 dpi. The experiments were repeated three times with the same results. Error bars represent standard deviations of data from three replicates. Normality and homogeneity of variance were checked before ANOVA; no data transformation was required. Lowercase indicates statistically significant differences among strains (Student–Newman–Keuls test, *p* < 0.05).

### All four velvet proteins are essential for asexual reproduction of *D. amygdali*

**Figure 4.**
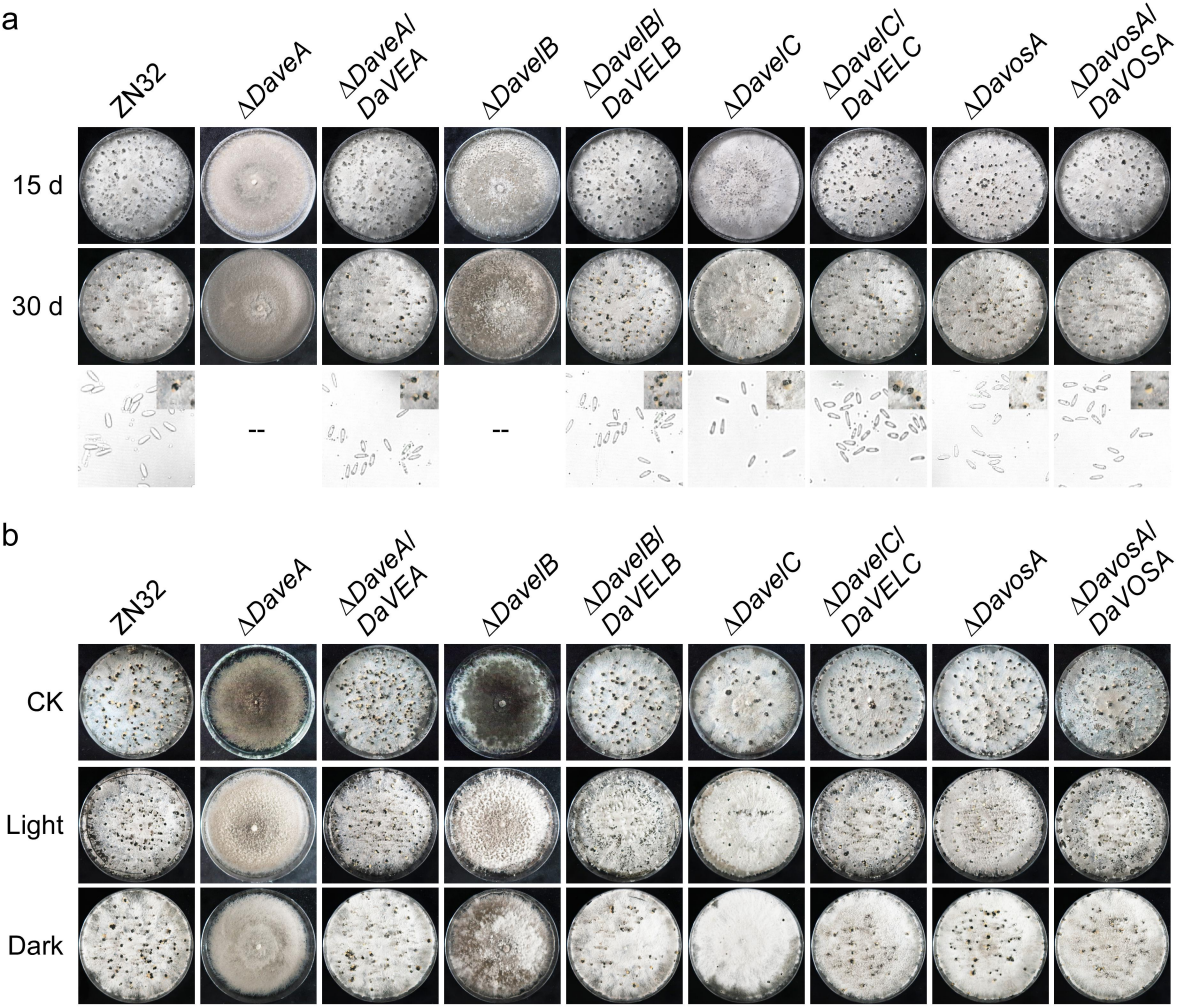
All four velvet proteins regulate the asexual reproduction in *D. amygdali*. (a) Hyphal blocks from ZN32, four single-gene velvet protein deletion mutants, and their complemented strains were inoculated onto PDA plates, and incubated at 25°C under a 12-h light/12-h dark photoperiod for 15 and 30 days to assess pycnidium and conidium formation, respectively. Conidial morphology was examined by light microscopy. (b) Strains were cultured under three distinct lighting regimes: continuous light, continuous darkness, and a 12-h light/12-h dark photoperiod (CK), for 30 days at 25°C. The experiments were repeated three times with the same results.

Given that conidia serve as the primary inoculum for field transmission and infection, we investigated the roles of velvet proteins in asexual reproduction. Wild-type strain ZN32, Δ*DaveA*, Δ*DavelB*, Δ*DavelC*, and Δ*DavosA* mutants, along with their complemented strains, were cultured on PDA plates at 25°C under a 12-h light/12-h dark photoperiod for 15 and 30 days. After 15 days, ZN32, Δ*DavosA*, and all complemented strains produced abundant, spherical, uncoated, and black pycnidia. However, no pycnidia or conidia were observed in Δ*DaveA* or Δ*DavelB* mutants at 15 days post-inoculation (dpi), and this developmental arrest persisted through 30 dpi. Besides that, Δ*DavelC* mutants formed fewer and smaller pycnidia, approximately 50% reduced (Figure 4(a) and Table 1). Conidial morphology was indistinguishable among all strains, except Δ*DaveA* or Δ*DavelB*, which produced no conidia whatsoever (Figure 4(a)).

Previous studies have reported that VeA-mediated regulation of fungal development is light-dependent [49]. To determine whether velvet proteins modulate pycnidia formation in a light-responsive manner, Δ*DaveA*, Δ*DavelB*, Δ*DavelC*, and Δ*DavosA* mutants were cultured at 25°C for 30 days under continuous light or continuous darkness, respectively; 12-h light/12-h dark photoperiod used as the control. Neither Δ*DaveA* nor Δ*DavelB* mutants produced any pycnidia or conidia under either light condition. Δ*DavelC* mutants consistently formed more fewer and smaller pycnidia under continuous light or darkness. Besides that, the pycnidia produced by Δ*DavosA* mutants under full light were smaller, but the shape was normal; under total darkness, normal-shaped but fewer pycnidia were produced (Figure 4(b) and Table 2). The results demonstrate that DaVeA, DaVelB, DaVelC, and DaVosA each contribute non-redundantly to asexual reproduction in *D. amygdali*, within DaVelC and DaVosA uniquely integrating photoperiod into this process.

**Table 2.** Pycnidia formation in *D. amygdali* single-gene velvet protein deletion mutants under different photoperiod at 30 days post-inoculation.

Strain	Number of pycnidia		
	12 h/12 h Light and dark cycle	Continuous light	Continuous dark
ZN32	133 ± 6.2a	138 ± 6.2a	128 ± 7.5a
Δ*DaveA*	0c	0c	0d
Δ*DaveA/DaVEA*	123 ± 3.5a	133 ± 5.7a	122 ± 8.6a
Δ*DavelB*	0c	0c	0d
Δ*DavelB/DaVELB*	132 ± 3.7a	137 ± 5.4a	126 ± 7.4a
Δ*DavelC*	62 ± 4.1b	57 ± 5.8b	19 ± 2.5c
Δ*DavelC/DaVELC*	129 ± 6.5a	131 ± 8.6a	123 ± 5.7a
Δ*DavosA*	132 ± 12.5a	129 ± 11.0a	96 ± 6.0b
Δ*DavosA/DaVOSA*	131 ± 6.2a	133 ± 4.0a	124 ± 4.4a

Testes strains were incubated at 25°C for 30 days under different photoperiod: 12 h/12 h light and dark cycle, continuous light, and continuous dark. The experiments were repeated three times with the same results. Normality and homogeneity of variance were checked before ANOVA; no data transformation was required. Lowercase represents statistically significant differences among strains (Student-Newman-Keuls test, *p* < 0.05).

### DaVeA and DaVelB are required for full virulence of *D. amygdali*

**Figure 5.**
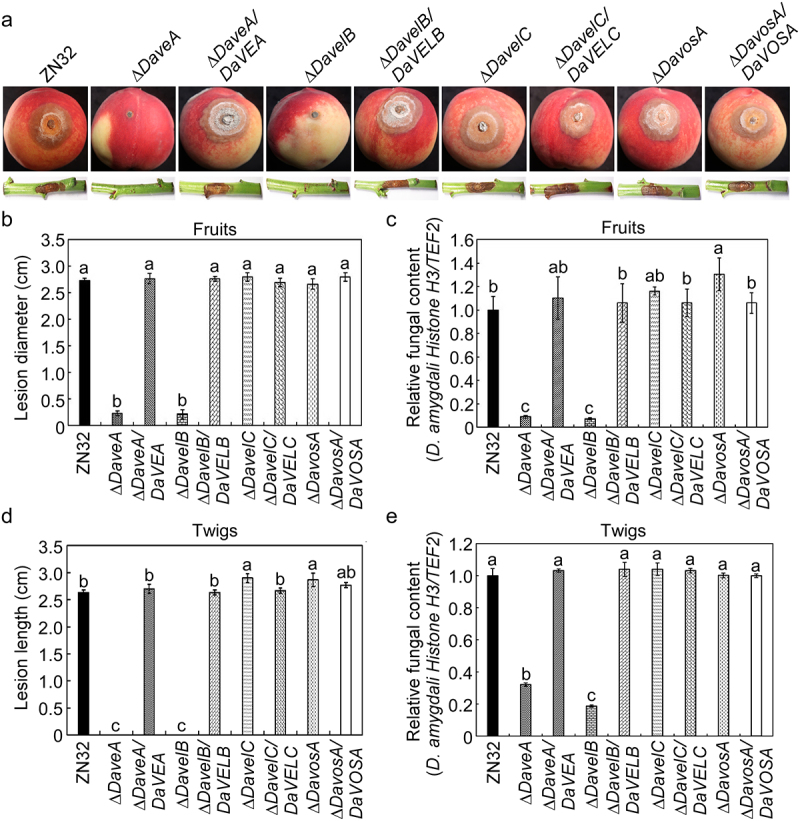
DaVeA and DaVelB are required for full virulence of *D. amygdali*. (a) Nine-millimeter hyphal blocks from the tested strains were inoculated onto surface-sterilized, wounded peach fruits or twigs, respectively. Inoculated tissues were incubated at 28°C within a 12-h light/12-h dark photoperiod under 90% relative humidity. Fruit lesions were observed at 5 days post-inoculation (dpi); twig lesions were observed at 7 dpi. (b) Lesion lengths on peach fruits were measured at 5 dpi. (c) Fungal biomass in infected peach fruit tissue was quantified by qRT-PCR using the *D. amygdali* histone H3-encoding gene (*DaH3*), and the *P. persica* translation elongation factor 2 (*PpTEF2*) was used to normalize different samples. (d) Lesion lengths on infected peach twigs were measured 7 dpi. (e) Fungal biomass in infected peach twig tissue was quantified by qRT-PCR. The experiments were repeated three times with the same results. Error bars represent standard deviations of data from three replicates. Normality and homogeneity of variance were checked before ANOVA; no data transformation was required. Lowercase indicates statistically significant differences (Student–Newman–Keuls test, *p* < 0.05).

We further assessed the function of velvet proteins to the virulence of *D. amygdali*. Sterilized, wounded peach fruits and twigs were inoculated with the wild-type strain ZN32, Δ*DaveA*, Δ*DavelB*, Δ*DavelC*, and Δ*DavosA* mutants, and their complemented strains. Then, incubated at 28°C under 90% RH and a 12 h/12 h light/dark cycle. Found that ZN32, Δ*DavelC*, Δ*DavosA* mutants, and all complemented strains induced necrotic lesions averaging ~2.7 cm in diameter on peach fruits at 5 dpi; however, there were only minute lesions (~0.25 cm) caused by Δ*DaveA* and Δ*DavelB* mutants (Figure 5(a,b)). Fungal biomass was quantified via DNA-based qRT-PCR targeting the conserved histone *DaH3* gene. Results showed that the relative fungal contents inoculated with Δ*DaveA* and Δ*DavelB* mutants were only one-tenth of the wild type ZN32 and complemented strains, however, Δ*DavelC* and Δ*DavosA* mutants exhibited no significant reduction (Figure 5(c)). Comparable lesion length and biomass defects were observed on peach twigs at 7 dpi (Figure 5(a,d,e)). In addition, we also inoculated above strains on wounded apple fruits, and observed reduced pathogenicity inoculated with Δ*DaveA* and Δ*DavelB* mutants, but not in Δ*DavelC* or Δ*DavosA* mutants (Figure S2). Therefore, these results suggest that DaVeA and DaVelB are required for full *D. amygdali* virulence, whereas DaVelC and DaVosA are not.

### DaVeA and DaVelB negatively regulate melanin production of *D. amygdali*

**Figure 6.**
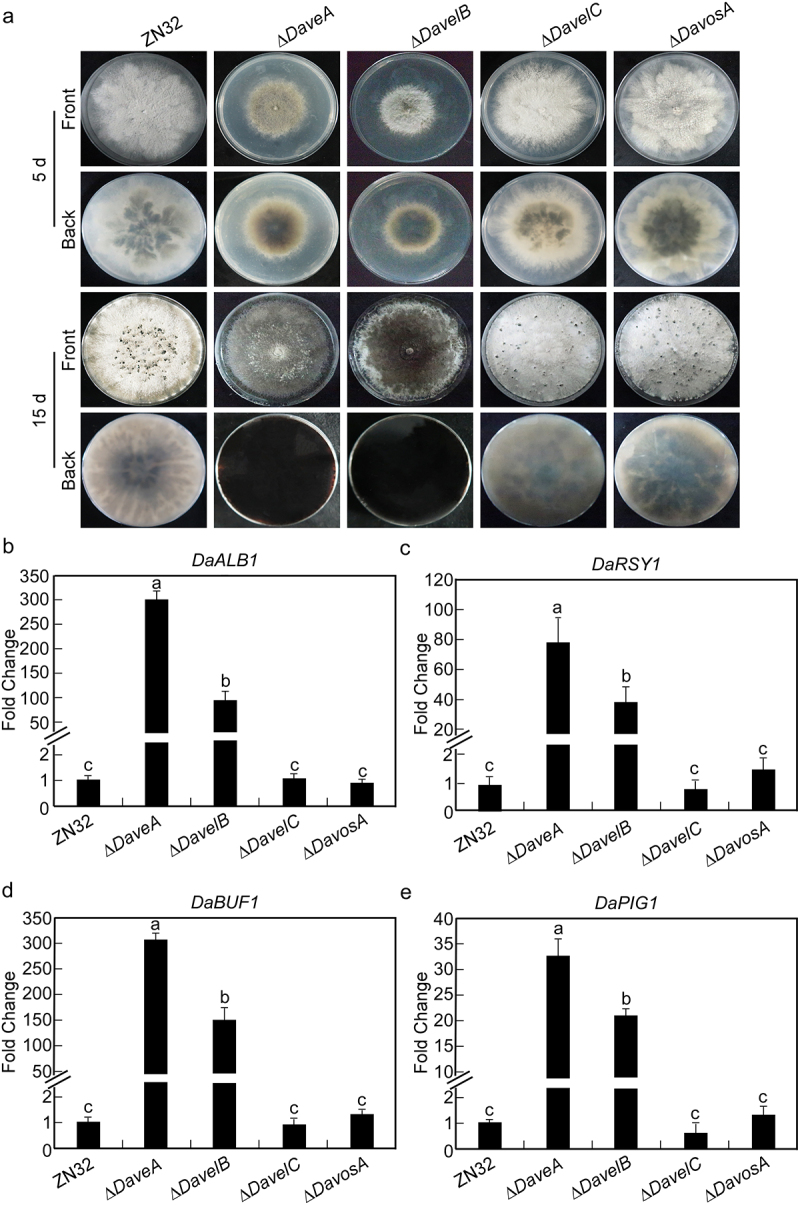
DaVeA and DaVelB negatively regulate melanin biosynthesis in *D. amygdali*. (a) ZN32 and single-gene velvet protein deletion mutants were cultured on potato dextrose agar (PDA) plates at 25°C under a 12-h light/12-h dark photoperiod for 5 and 15 days. (b-e) Transcript levels of melanin biosynthesis genes, including*DaALB1,DaRSY1,DaBUF1*, and*DaPIG1*, determined by qRT-PCR in ZN32 and single-gene velvet protein deletion mutants, respectively. The*D. amygdali*histone H3-encoding gene (*DaH3*) served as the endogenous reference gene. The experiments were repeated three times with the same results. Error bars represent standard deviations of data from three replicates. Normality and homogeneity of variance were checked before ANOVA; no data transformation was required. Lowercase indicates statistically significant differences among strains (Student–Newman–Keuls test, *p* < 0.05).

We further observed the melanin production of the single-gene velvet protein deletion mutants on PDA plates at 5 and 15 dpi. The results found that, in both Δ*DaveA* and Δ*DavelB* mutants, the melanin accumulation was significantly increased at the back of PDA plates relative to the wild-type strain ZN32, Δ*DavelC* and Δ*DavosA* mutants at 5 dpi, particularly after extended incubation for another 10 days (Figure 6(a)). In addition, we assessed transcript levels of four melanin biosynthesis‑related genes (*DaALB1*, *DaRSY1*, *DaBUF1*, and *DaPIG1*) in ZN32 strain and the deletion mutants using qRT-PCR. Expression of all four genes was significantly upregulated in Δ*DaveA* (30–300-fold) and Δ*DavelB* (20–150-fold) relative to ZN32 strain; no significant changes were detected in Δ*DavelC* or Δ*DavosA* mutants (Figure 6(b–e)). Indicating DaVeA and DaVelB negatively regulate the melanin production of *D. amygdali*.

### DaVeA and DaVelB regulate the expression of genes encoding cell wall- degrading enzymes (CWDEs) in *D. amygdali*

**Figure 7.**
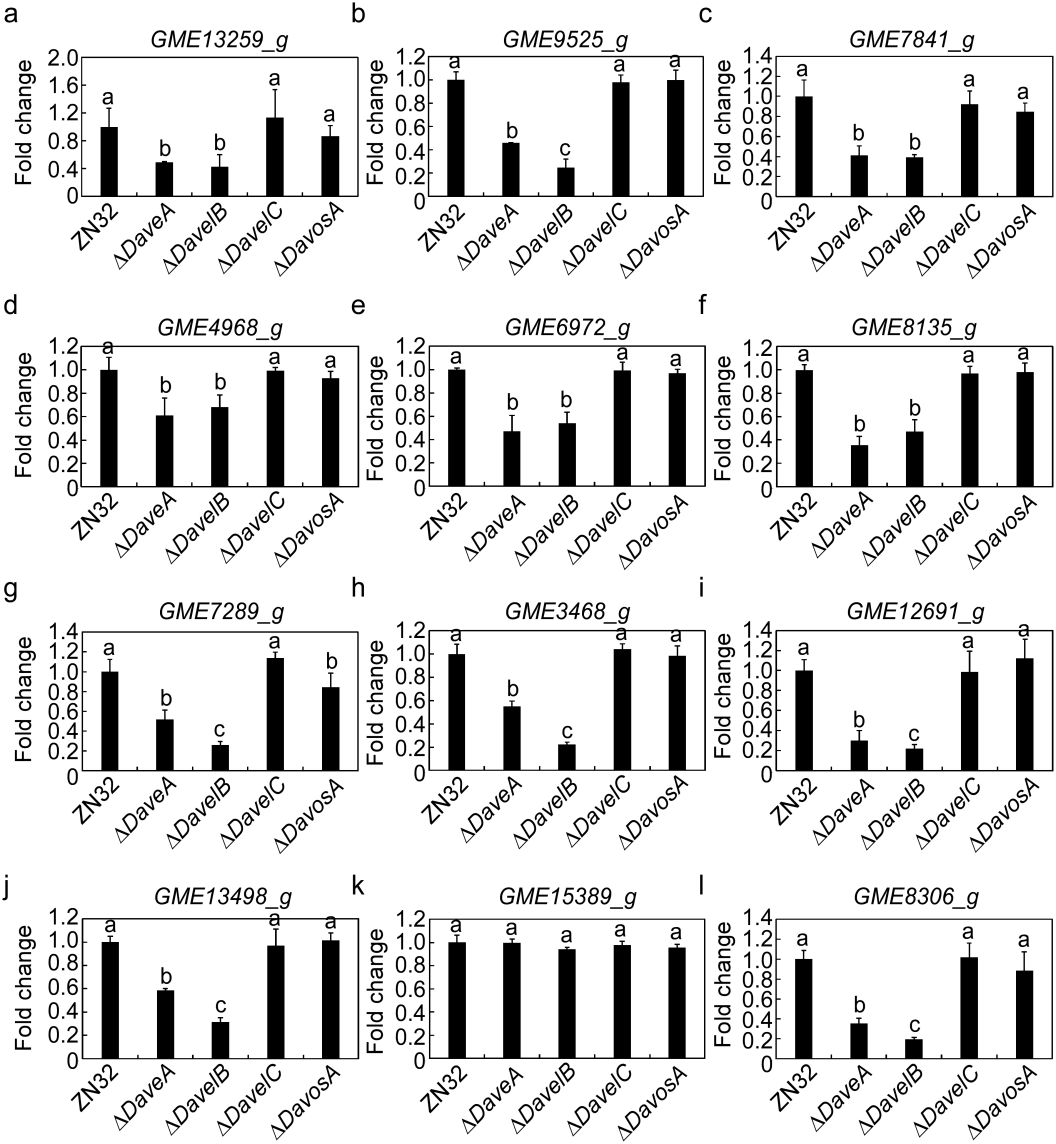
Expression of cell wall-degrading enzymes (CWDEs) genes in four *D. amygdali* single-gene velvet deletion mutants. (a-l) transcript levels of CWDEs-encoding genes were quantified in the wild-type strain ZN32 and four single-gene velvet deletion mutants by qRT-PCR. Pectinase-encoding genes(a-c), cellulase-encoding genes(d-f), hemicellulase-encoding genes(g-i), and lignin-modifying enzyme-encoding genes(j-l). The experiments were repeated three times with the same results. Error bars represent standard deviations of data from three replicates. Normality and homogeneity of variance were checked before ANOVA; no data transformation was required. Lowercase indicates statistically significant differences among strains (Student–Newman–Keuls test, *p* < 0.05).

CWDE expression might contribute to host tissue colonization and lesion expansion after wound-mediated entry. To determine whether velvet proteins in *D. amygdali* modulate CWDEs gene expression, we selected three representative genes: pectinases (GME13259_g, GME9525_g, GME7841_g), cellulases (GME4968_g, GME6972_g, GME8135_g), hemi-cellulases (GME7289_g, GME3468_g, GME12691_g), and lignin-modifying enzymes (GME13498_g, GME15389_g, GME8306_g). Transcript levels of these 12 genes were quantified in the ZN32 and single-gene velvet deletion mutants via qRT-PCR, respectively. Compared with ZN32 strain, 11 of the 12 target genes exhibited significant downregulation in both Δ*DaveA* and Δ*DavelB* mutants; in contrast, no significant changes were observed in either Δ*DavelC* or Δ*DavosA* mutants (Figure 7(a–l)). These findings suggest that DaVeA and DaVelB, rather than DaVelC or DavosA, are key transcriptional regulators of diverse CWDE-encoding genes.

### DaVeA and DaVelB regulate the expression of the fusicoccin biosynthetic gene cluster in *D. amygdali*

**Figure 8.**
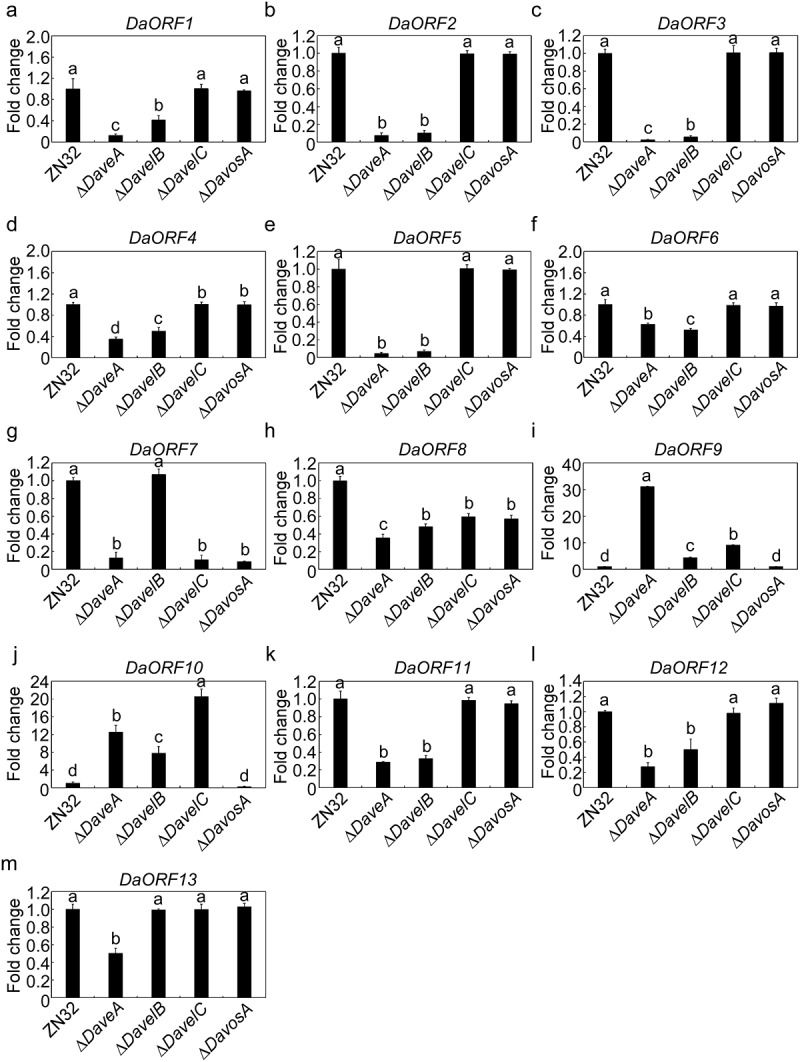
DaVeA and DaVelB positively regulate the expression of the fusicoccin biosynthetic gene cluster in *D. amygdali*. (a-m) Transcript levels of fusicoccin biosynthetic genes (*ORF1-ORF13*) were quantified in the wild-type strain ZN32 and four single-gene velvet protein deletion mutants by qRT-PCR. The experiments were repeated three times with the same results. Error bars represent standard deviations of data from three replicates. Normality and homogeneity of variance were checked before ANOVA; no data transformation was required. Lowercase indicates statistically significant differences among strains (Student–Newman–Keuls test, *p* < 0.05).

*D. amygdali* could produce fusicoccin, a kind of diterpene phytotoxin, that binds peach 14–3-3 proteins, thereby locking stomata in an open state and ultimately inducing premature leaf abscission [50,51]. Here, we detected the expression of 13 genes (*ORF1-ORF13*) which function in the fusicoccin biosynthetic pathway through qRT-PCR. The transcript levels of *ORF1-ORF8* and *ORF11-ORF13* were significantly downregulated in Δ*DaveA* mutants compared with that of in ZN32 strain; *ORF1-ORF6*, *ORF8*, and *ORF11-ORF12* were dramatically downregulated in Δ*DavelB* mutants. However, there was no significant transcript difference among Δ*DavelC*, Δ*DavosA* mutants and ZN32 strain (Figure 8(a–m)). These results demonstrate that DaVeA and DaVelB function as positive transcriptional regulators of the fusicoccin biosynthetic pathway in *D. amygdali*.

### Deletion of *DaVEA* or *DaVELB* affect the colony surface hydrophobicity of *D. amygdali*

To assess the roles of *D. amygdali* velvet proteins in colony surface hydrophobicity, the wild-type ZN32 and single-gene velvet deletion mutants were cultured on PDA plates for 5 days, and treated with sterile water or detergent solution for 3 min. Results found that ZN32, Δ*DavelC* and Δ*DavosA* mutants remained hydrophobic that water droplets retained spherical shape and did not spread when treated with the sterile water; however, droplets collapsed rapidly upon detergent solution application. In contrast, Δ*DaveA* and Δ*DavelB* mutants exhibited constitutive hydrophobicity which water droplets failed to collapse even after detergent solution treatment (Figure S3a). We further quantified transcript levels of two key hydrophobicity-encoding genes, *DaMHP1* and *DaPTH11*, using qRT-PCR. Expression of *DaMHP1* was upregulated to 5.0-fold in Δ*DaveA* and 4.5-fold in Δ*DavelB* mutants compared to ZN32; *DaPTH11* expression increased 4.0-fold in Δ*DaveA* and 3.0-fold in Δ*DavelB* mutants. No significant differences of either gene were detected in Δ*DavelC* or Δ*DavosA* mutants (Figure S3b-c), indicating that DaVeA and DaVelB negatively regulate surface hydrophobicity in *D. amygdali*.

### The complex interactions within velvet proteins in *D. amygdali*

**Figure 9.**
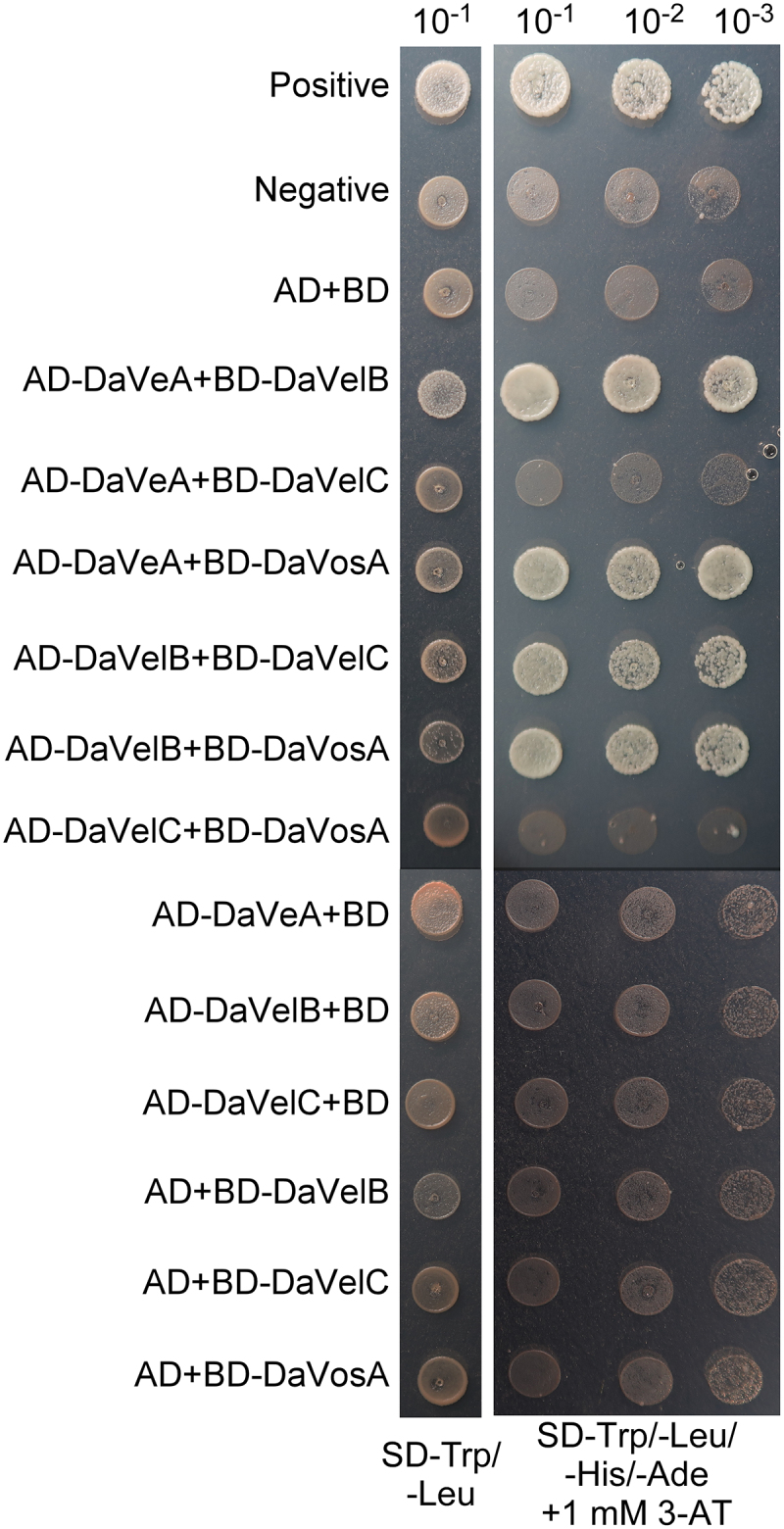
Yeast two-hybrid system to detect the interactions among four velvet proteins in *D. amygdali*. Yeast transformants expressing the bait and prey constructs of *DaVEA, DaVELB, DaVELC*, and *DaVOSA* were growth on synthetic dextrose medium (SD) lacking tryptophan (Trp) and leucine (leu) for 3 days, followed by photocopying onto SD–Trp/–leu/–his/–ade supplemented with 1 mM 3-amino-1,2,4-triazole (3-AT), after incubation at 28°C for another 3 days. Pair pGBKT7-53/pGADT7 was used as positive control; pGBKT7-Lam/pGADT7 pair set as negative control. The experiments were repeated three times with the same results.

Members of the velvet protein are known to engage in mutual physical interactions [36–38]. We performed yeast two-hybrid system to investigate the direct interaction among velvet proteins in *D. amygdali*. Results confirmed that DaVeA interacted with DaVelB and DaVosA, but not with DaVelC; conversely, DaVelB interacted with DaVeA, DaVelC, and DaVosA (Figure 9). These results reveal a partially overlapping yet distinct interaction topology within the *D. amygdali* velvet proteins.

### DaVeA and DaVelB contribute to stress response in *D. amygdali*

**Figure 10.**
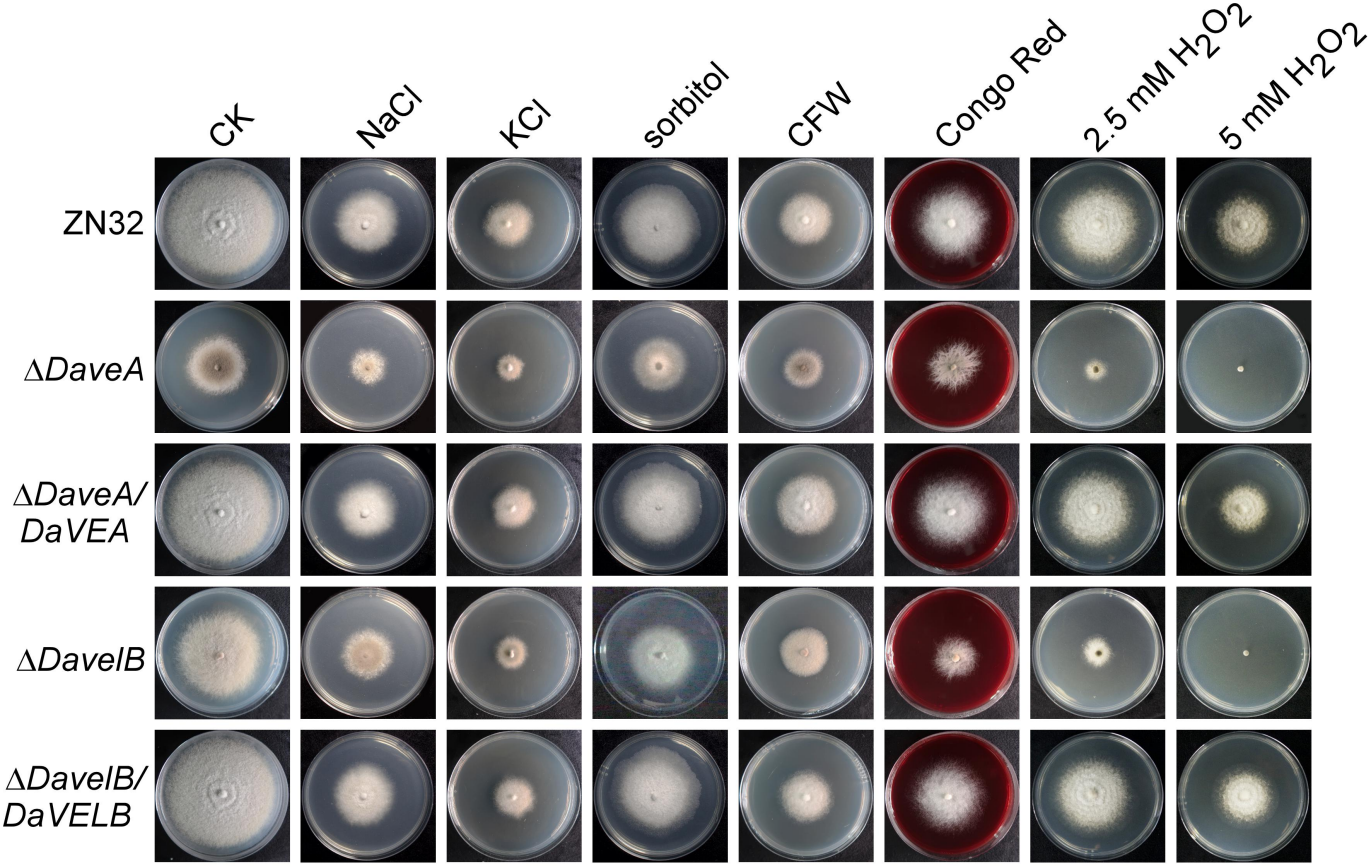
DaVeA and DaVelB contribute to stress response in *D. amygdali*. Wild-type ZN32, Δ*DaveA*, Δ*DavelB*, and the corresponding complemented strains were inoculated on complete medium (CM) plates supplemented with various stress-inducing agents, including 0.4 M NaCl, 0.6 M KCl, 1.0 M sorbitol, 400 µg/mL calcofluor white (CFW), 400 µg/mL Congo Red, 2.5 mM and 5.0 mM H_2_O_2_. Strains inoculated on the CM plates without any stressors were used as the control. Photographs were taken after 5 days. The experiments were repeated three times with the same results.

To determine whether DaVeA and DaVelB regulate abiotic stress tolerance, wild-type ZN32, Δ*DaveA*, Δ*DavelB* mutants, and their corresponding complemented strains were inoculated on CM plates supplemented with osmotic stress agents (0.4 M NaCl, 0.6 M KCl, and 1.0 M sorbitol), cell wall-perturbing agents (400 μg/mL calcofluor white [CFW] and 400 μg/mL Congo Red), and oxidative stress agents (2.5 mM and 5.0 mM H_2_O_2_), respectively. Both Δ*DaveA* and Δ*DavelB* mutants exhibited significantly increased sensitivity compared with ZN32 and complemented strains under NaCl, KCl, CFW, and oxidative stress conditions (Figure 10 and Table 3). Notably, the Δ*DaveA* mutants exhibited significantly enhanced tolerance to Congo Red, whereas the Δ*DavelB* mutants showed no statistically significant difference. All strains showed similar sensitivity to sorbitol (Figure 10 and Table 3). These results suggest that DaVeA and DaVelB play distinct yet overlapping roles in mediating responses to diverse abiotic stresses in *D. amygdali*.

**Table 3.** Sensitivity analysis of *D. amygdali* single-gene velvet protein deletion mutants to various abiotic stressors.

Strain	Growth inhibition rate (%)						
	0.4 M NaCl	0.6 M KCl	1.0 M sorbitol	400 μg/mL Congo Red	400 μg/mL CFW	2.5 mM H_2_O_2_	5 mM H_2_O_2_
ZN32	40.1 ± 2.0c	57.3 ± 1.4b	35.4 ± 2.9a	51.4 ± 1.4a	34.5 ± 0.7c	1.6 ± 0.7c	2.8 ± 0.7c
Δ*DaveA*	60.6 ± 1.2a	62.1 ± 0.7a	37.9 ± 2.1a	43.5 ± 0.7b	41.6 ± 1.2b	10.6 ± 1.5a	18.7 ± 1.9a
Δ*DaveA*/*DaVEA*	41.9 ± 1.2c	57.3 ± 0.7b	35.4 ± 1.4a	51.4 ± 0.7a	34.5 ± 0.7c	1.9 ± 0.7c	2.8 ± 0.7c
Δ*DavelB*	56.8 ± 1.6b	64.4 ± 2.7a	35.1 ± 4.5a	55.2 ± 1.6a	51.6 ± 2.4a	7.2 ± 1.1b	13.6 ± 1.2b
Δ*DavelB*/*DaVELB*	42.8 ± 2.2c	58.3 ± 1.1b	36.9 ± 2.9a	50.8 ± 3.9a	33.9 ± 2.2c	1.7 ± 0.7c	2.8 ± 0.7c

Tested strains were inoculated on complete medium (CM) supplemented with different stressors and cultured under alternating 12 h/12 h light and dark cycle at 25°C for 5 days, respectively. These strains were also inoculated on CM medium without any stressors as a control. The final concentrations used were 0.4 M NaCl, 0.6 M KCl, 1.0 M sorbitol, 400 μg/mL Congo Red, 400 μg/mL CFW, 2.5 mM and 5 mM H_2_O_2_. Growth inhibition rate = (diameter of untreated strain – diameter of treated strain)/diameter of untreated strain × 100%. The experiments were repeated three times with the same results. Normality and homogeneity of variance were checked before ANOVA; no data transformation was required. Lowercase represents statistically significant differences among strains (Student–Newman–Keuls test, *p* < 0.05).

## Discussion

In this study, we identified four velvet proteins in peach shoot blight fungus *D*. *amygdali* and systematically characterized their functional roles. DaVeA and DaVelB are multifunctional regulators required for vegetative growth, melanin biosynthesis, asexual reproduction, pathogenicity, expression of genes encoding cell wall-degrading enzymes, surface hydrophobicity, and fusicoccin biosynthetic gene cluster, and responses to diverse abiotic stresses. In contrast, DaVelC and DaVosA exhibit a more restricted functional scope, being specifically involved in asexual reproduction.

Deletion of *DaVEA* or *DaVELB*, unlike deletion of *DaVELC* or *DaVOSA*, resulted not only in altered colony morphology and reduced radial growth of *D. amygdali* (Figure 3). This observation aligns with established roles of VeA and VelB as positive regulators of vegetative growth in *A. parasiticus* [24], *F. graminearum* [39], *F. oxysporum* [40] and *C. sativus* [27]. In contrast, VeA and VelB orthologs in *V. mali*, despite sharing high sequence homology with DaVeA and DaVelB, do not influence vegetative growth [28]. Notably, functional divergence is also evident in *B. cinerea* that BcVelB promotes vegetative growth, whereas BcVeA appears dispensable for this process [23]. Indicated that VeA and VelB are critical for fungal vegetative growth and underscore species-specific functional evolution of velvet proteins.

Deletion of *DaVEA* or *DaVELB* completely abolished pycnidium and conidium formation in *D. amygdali* (Figure 4(a)). To our knowledge, it was the first report demonstrating that knock out a velvet protein entirely abolishes the fungal asexual reproductive capacity. Besides that, DaVelC and DaVosA modulate the *D. amygdali* asexual reproduction in a response photoperiod-dependent manner (Figure 4(b)). Velvet proteins exert diverse, often opposing, regulatory roles in asexual reproduction across plant pathogenic fungi. In *V*. *mali* [28], *B*. *cinerea* [23] and *F*. *graminearum* [39], VeA and VelB function as negative regulators of conidiation; conversely, they act as positive regulators in *M. oryzae* [42] and *A. parasiticus* [24]. Functional divergence is also observed among paralogs within the same species, VeA represses conidiation whereas VelB promotes it in *A. nidulans* [36,52]. Similarly, in *P. chrysogenum*, PcVeA and PcVelC negatively regulate conidiation, whereas PcVelB and PcVosA positively regulate it [44]. Collectively, these findings underscore that the conserved velvet proteins play evolutionarily conserved roles in governing fungal asexual reproduction. We hypothesize that in *D. amygdali*, unlike in other fungi, no functional redundancy exists among velvet proteins for activating the conidiation cascade.

Virulence assays showed that DaVeA and DaVelB are required for the pathogenicity of *D. amygdali*, whereas DaVelC and DaVosA are dispensable for this process (Figure 5). These results are consistent with findings in *V*. *mali* [28]. Supporting their broad functional conservation, VeA and VelB orthologs are also involved in full virulence in *F. graminearum* [39], *B*. *cinerea* [23] and *F. oxysporum* [40]. In contrast, functional requirements differ markedly in *M. oryzae*, MoVeA and MoVelC, but not MoVelB or MoVosA, are critical for pathogenicity [42]. Notably, in *Cochliobolus sativus*, all four velvet proteins, CsVeA, CsVelB, CsVelC, and CsVosA contribute to virulence [27,38]. As an opportunistic pathogen, *D. amygdali* relies primarily on CWDEs and host-specific toxins, such as fusicoccin, for successful infection. Transcript levels of genes encoding pectinases, cellulases, hemicellulases, lignin-modifying enzymes, and fusicoccin biosynthetic enzymes were nearly uniformly downregulated in Δ*DaveA* or Δ*DavelB* mutants (Figure 7–8). Intriguingly, in *V. mali*, *VmVEA* and *VmVELB* deletion impaired pectinase activity, with no measurable impact on hemicellulase, cellulase, or ligninase-related gene expression [28]. These cross-species comparisons reveal that velvet proteins, despite structural conservation, mediate pathogenicity through divergent molecular mechanisms, adapted to the specific infection strategies of their respective hosts. We propose that DaVeA and DaVelB coordinate the expression of a broad “pathogenicity arsenal” in *D. amygdali* by directly or indirectly binding to the promoters of CWDE and fusicoccin biosynthetic genes, possibly in complex with DaLaeA. The lack of virulence defect in Δ*DalaeA* (data not shown) suggests that DaVeA/DaVelB may also function through LaeA-independent pathways, such as recruiting other co-activators or modulating chromatin accessibility via histone modifications.

Numerous studies have established that velvet proteins play pivotal roles in the biosynthetic regulation of melanin, mycotoxins, and other fungal secondary metabolites [53]. Deletion of *DaVEA* or *DaVELB* resulted in a markedly darker colony pigmentation of *D*. *amygdali*, consistent with dysregulated melanin accumulation. Expression of key melanin biosynthesis-related genes (*ALB1*, *RSY1*, *BUF1*, and *PIG1*) were significantly upregulated in both Δ*DaveA* and Δ*DavelB* mutants compared with that of in the ZN32, Δ*DavelC* and Δ*DavosA* mutants (Figure 6). These findings align with reports in *V*. *mali* and *B. cinerea*, where VeA and VelB act as negative regulators of melanin production [23,28]. Similar repressive functions are observed for MgVeA in *M. graminicola* and for CsVeA, CsVelB, and CsVosA in *C. sativus* [26,27,38]. In *Aspergillus* species, VeA is a well-characterized positive regulator of aflatoxin biosynthesis in *A. nidulans*, *A. flavus* and *A. parasiticu* [24,29,30]. Furthermore, VeA and VelB positively regulate beauvericin production in *F*. *oxysporum* and deoxynivalenol (DON) in *F. graminearum* [39,40]. Critically, our study revealed that DaVeA and DaVelB might be positive regulators of fusicoccin biosynthesis in *D. amygdali*, extending the functional conservation of velvet proteins to host-specific phytotoxin production.

In *A. nidulans*, VeA can interact with VelB and LaeA, and form heterotrimer to co-regulate the fungal development [54]. However, our results showed that DaLaeA can not only interact with DaVeA but also with DaVelB (Figure S4). Unexpectedly, deletion of *DaLAEA* showed no changes in virulence compared with the wild-type (data not shown). Previous reports have indicated that members of the velvet family proteins can also form complexes, including VelB/VosA, VelB/VelB, VelC/VosA, and VelC/VeA [55–57]. Our research showed DaVeA could interact with DaVelB and DaVosA but DaVelC, whereas DaVelB could interact with DaVeA, DaVelC, and DaVosA in *D. amygdali*.

In conclusion, this study provides a comprehensive functional characterization of the four velvet proteins in *D. amygdali*. Our findings expand the functional repertoire of velvet proteins across plant pathogenic fungi and elucidate key molecular determinants underlying the pathogenesis of peach shoot blight.

## Supplementary Material

Supporting information.docx

## Data Availability

The source data of this study are openly available at https://doi.org/10.6084/m9.figshare.31146700.v2 [54].
