# Supplementary material for: Characterization of velvet family proteins regulating fungal development and virulence in the peach shoot blight fungus Diaporthe amygdali
Source: Virulence. 2026 Aug 14;17(1):2715219. doi: 10.1080/21505594.2026.2715219 (PMC13479829; doi:10.1080/21505594.2026.2715219)
Supplement: Supplementary file 1 [file KVIR_A_2715219_SM3944.docx]

**Figure Legends and Tables**

**Figure 1****.** Phylogenetic analysis of velvet proteins and domain prediction. (a) A maximum-likelihood phylogenetic tree was constructed using the neighbor-joining method by MEGA11. Pd: *Penicillium digitatum*; Pc: *Penicillium citrinum*; Ap: *Aspergillus parasiticus*; An: *Aspergillus nidulans*; Zt: *Zymoseptoria tritici*; Fo: *Fusarium oxysporum*; Fg: *Fusarium graminearum*; Nc: *Neurospora crassa*; Mo: *Magnaporthe oryzae*; Vm: *Valsa mali*; Da: *Diaporthe amygdali*. (b) Domain of velvet proteins in *D. amygdali* were predicted using the Conserved Domain Database (CDD) v3.20 via NCBI’s Batch CD-Search tool.

**Figure 2.** Transcriptional profiling of velvet protein-encoding genes across developmental and infection stages in *D. amygdali*. (a-d) Expression levels of velvet genes, including *DaVEA*, *DaVELB*, *DaVELC*, and *DaVOSA*, were quantified by qRT-PCR in vegetative hyphae (HY), pycnidia (PY), conidia (Co), and infected peach twigs at 12, 24, 36, 48, and 72 hours post-inoculation (hpi). The experiments were repeated three times with the same results. Error bars represent standard deviations of data from three replicates. Normality and homogeneity of variance were checked before ANOVA; no data transformation was required. Lowercase indicates statistically significant differences (Student–Newman–Keuls test, *P* < 0.05).

**Figure 3.** DaVeA and DaVelB regulate vegetative growth in *D. amygdali*. Wild-type, single-gene velvet protein deletion mutants, and their complemented strains were cultured on potato dextrose agar (PDA), complete medium (CM), oatmeal agar (OM), and alfalfa decoction–Czapek medium (AC) at 25°C under a 12-h light/12-h dark photoperiod for 5 days, respectively. The experiments were repeated three times with the same results.

**Figure** **4.** All four velvet proteins regulate the asexual reproduction in *D. amygdali*. (a)Hyphal blocks from ZN32, four single-gene velvet protein deletion mutants, and their complemented strains were inoculated onto PDA plates, and incubated at 25 °C under a 12-h light/12-h dark photoperiod for 15 and 30 days to assess pycnidium and conidium formation, respectively. Conidial morphology was examined by light microscopy. (b) Strains were cultured under three distinct lighting regimes: continuous light, continuous darkness, and a 12-h light/12-h dark photoperiod (CK), for 30 days at 25°C. The experiments were repeated three times with the same results.

**Figure 5.** DaVeA and DaVelB are required for full virulence of *D. amygdali*. (a) Five-millimeter hyphal blocks from the tested strains were inoculated onto surface-sterilized, wounded peach fruits or twigs. Inoculated tissues were incubated at 28°C within a 12-h light/12-h dark photoperiod under 90% relative humidity. Fruit lesions were observed at 5 days post-inoculation (dpi); twig lesions were observed at 7 dpi. (b) Lesion lengths on peach fruits were measured at 5 dpi. (c) Fungal biomass in infected peach fruit tissue was quantified by qRT-PCR using the *D. amygdali* histone H3-encoding gene (*DaH3*), and the *P. persica* translation elongation factor 2 (*PpTEF2*) was used to normalize different samples. (d) Lesion lengths on infected peach twigs were measured 7 dpi. (e) Fungal biomass in infected peach twig tissue was quantified by qRT-PCR. The experiments were repeated three times with the same results. Error bars represent standard deviations of data from three replicates. Normality and homogeneity of variance were checked before ANOVA; no data transformation was required. Lowercase indicates statistically significant differences (Student–Newman–Keuls test, *P* < 0.05).

**Figure 6.** DaVeA and DaVelB negatively regulate melanin biosynthesis in *D. amygdali*.

(a) ZN32 and single-gene velvet protein deletion mutants were cultured on potato dextrose agar (PDA) plates at 25°C under a 12-h light/12-h dark photoperiod for 5 and 15 days. (b-e) Transcript levels of melanin biosynthesis genes, including *DaALB1*, *DaRSY1*, *DaBUF1*, and *DaPIG1*, determined by qRT-PCR in ZN32 and single-gene velvet protein deletion mutants, respectively. The *D. amygdali* histone H3-encoding gene (*DaH3*) served as the endogenous reference gene. The experiments were repeated three times with the same results. Error bars represent standard deviations of data from three replicates. Normality and homogeneity of variance were checked before ANOVA; no data transformation was required. Lowercase indicates statistically significant differences among strains (Student–Newman–Keuls test, *P* < 0.05).

**Figure 7.** Expression of cell wall-degrading enzymes (CWDEs) genes in four *D. amygdali* single-gene velvet deletion mutants. (a-l) Transcript levels of CWDEs-encoding genes were quantified in the wild-type strain ZN32 and four single-gene velvet deletion mutants by qRT-PCR. Pectinase-encoding genes(a-c), cellulase-encoding genes(d-f), hemicellulase-encoding genes(g-i), and lignin-modifying enzyme-encoding genes(j-l). The experiments were repeated three times with the same results. Error bars represent standard deviations of data from three replicates. Normality and homogeneity of variance were checked before ANOVA; no data transformation was required. Lowercase indicates statistically significant differences among strains (Student–Newman–Keuls test, *P* < 0.05).

**Figure** **8.** DaVeA and DaVelB positively regulate the expression of the fusicoccin biosynthetic gene cluster in *D. amygdali*. (a-m) Transcript levels of fusicoccin biosynthetic genes (*ORF1*-*ORF13*) were quantified in the wild-type strain ZN32 and four single-gene velvet protein deletion mutants by qRT-PCR. The experiments were repeated three times with the same results. Error bars represent standard deviations of data from three replicates. Normality and homogeneity of variance were checked before ANOVA; no data transformation was required. Lowercase indicates statistically significant differences among strains (Student–Newman–Keuls test, *P* < 0.05).

**Figure** **9.** Yeast two-hybrid system to detect the interactions among four velvet proteins in *D. amygdali*. Yeast transformants expressing the bait and prey constructs of *DaVEA*, *DaVELB*, *DaVELC*, and *DaVOSA* were growth on synthetic dextrose medium (SD) lacking tryptophan (Trp) and leucine (Leu) for 3 days, followed by photocopying onto SD–Trp/–Leu/–His/–Ade supplemented with 1 mM 3-amino-1,2,4-triazole (3-AT), after incubation at 28°C for another 3 days. Pairs pGBKT7-53/pGADT7 were used as positive control; pGBKT7-Lam/pGADT7 set as negative control. The experiments were repeated three times with the same results.

**Figure** **10.** DaVeA and DaVelB contribute to stress response in *D. amygdali*. Wild-type ZN32, Δ*DaveA*, Δ*DavelB*, and the corresponding complemented strains were inoculated on complete medium (CM) plates supplemented with various stress-inducing agents, including 0.4 M NaCl, 0.6 M KCl, 1.0 M sorbitol, 400 µg/mL calcofluor white (CFW), 400 µg/mL Congo Red, 2.5 mM and 5.0 mM H₂O₂. Strains inoculated on the CM plates without any stressors were used as the control. Photographs were taken after 5 days. The experiments were repeated three times with the same results.

**Table 1** **Phenotypic measurements of *D. amygdali*** **single-gene velvet protein deletion mutants.**

| Strain | Colony diameter（cm） | | | | Number of pycnidia | |
| --- | --- | --- | --- | --- | --- | --- |
|  | PDA | CM | OM | AC | 15 d | 30 d |
| ZN32 | 8.1 ± 0.1a | 8.1 ± 0.1a | 6.2 ± 0.1a | 6.4 ± 0.1a | 130 ± 1.5a | 137 ± 7.6a |
| Δ*DaveA* | 5.1 ± 0.3c | 4.8 ± 0.1c | 4.5 ± 0.2b | 3.2 ± 0.1c | 0c | 0c |
| Δ*DaveA*/*DaVEA* | 8.1 ± 0.1a | 8.1 ± 0.2a | 6.1 ± 0.1a | 6.4 ± 0.1a | 128 ± 4.0a | 131 ± 11.0a |
| Δ*DavelB* | 6.1 ± 0.2b | 6.4 ± 0.1b | 4.0 ± 0.1c | 4.9 ± 0.1b | 0c | 0c |
| Δ*DavelB*/*DaVELB* | 8.1 ± 0.1a | 8.0 ± 0.1a | 6.1 ± 0.1a | 6.2 ± 0.2a | 128 ± 3.1a | 133 ± 5.7a |
| Δ*DavelC* | 8.0 ± 0.1a | 8.1 ± 0.1a | 6.1 ± 0.1a | 6.4 ± 0.1a | 61 ± 2.1b | 57 ± 7.1b |
| Δ*DavelC*/*DaVELC* | 7.7 ± 0.1a | 8.1 ± 0.1a | 6.1 ± 0.1a | 6.5 ± 0.1a | 132 ± 2.1a | 136 ± 8.6a |
| Δ*DavosA* | 8.0 ± 0.1a | 8.1 ± 0.1a | 6.1 ± 0.2a | 6.4 ± 0.1a | 133 ± 5.5a | 129 ± 11.0a |
| Δ*DavosA*/*DaVOSA* | 8.0 ± 0.1a | 8.0 ± 0.2a | 6.2 ± 0.1a | 6.5 ± 0.1a | 130 ± 4.0a | 133 ± 4.0a |

Colony diameters of all test strains were measured on potato dextrose agar (PDA), complete medium (CM), oatmeal agar (OM), and alfalfa decoction–czapek agar (AC) at 25°C under a 12-h light/12-h dark photoperiod at 5 days post-inoculation. The number of pycnidia of tested strains was measured at 15 and 30 dpi. The experiments were repeated three times with the same results. Error bars represent standard deviations of data from three replicates. Normality and homogeneity of variance were checked before ANOVA; no data transformation was required. Lowercase indicates statistically significant differences among strains (Student–Newman–Keuls test, *P* < 0.05).

**Table** **2** **Pycnidia formation in *D. amygdali* s****ingle-gene velvet protein deletion mutants under different** **photoperiod at 30 days post-inoculation.**

| Strain | Number of pycnidia | | |
| --- | --- | --- | --- |
|  | 12 h/12 h Light and dark cycle | Continuous light | Continuous dark |
| ZN32 | 133 ± 6.2a | 138 ± 6.2a | 128 ± 7.5a |
| Δ*DaveA* | 0c | 0c | 0d |
| Δ*DaveA/DaVEA* | 123 ± 3.5a | 133 ± 5.7a | 122 ± 8.6a |
| Δ*DavelB* | 0c | 0c | 0d |
| Δ*DavelB/DaVELB* | 132 ± 3.7a | 137 ± 5.4a | 126 ± 7.4a |
| Δ*DavelC* | 62 ± 4.1b | 57 ± 5.8b | 19 ± 2.5c |
| Δ*DavelC/DaVELC* | 129 ± 6.5a | 131 ± 8.6a | 123 ± 5.7a |
| Δ*DavosA* | 132 ± 12.5a | 129 ± 11.0a | 96 ± 6.0b |
| Δ*DavosA/DaVOSA* | 131 ± 6.2a | 133 ± 4.0a | 124 ± 4.4a |

Testes strains were incubated at 25°C for 30 days under different photoperiod: 12 h/12 h light and dark cycle, continuous light, and continuous dark. The experiments were repeated three times with the same results. Normality and homogeneity of variance were checked before ANOVA; no data transformation was required. Lowercase represents statistically significant differences among strains (Student-Newman-Keuls test, *P* < 0.05).

**Table** **3** **Sensitivity analysis of *D. amygdali* single-gene velvet protein deletion mutants to various abiotic stressors.**

| Strain | Growth inhibition rate (%) | | | | | | |
| --- | --- | --- | --- | --- | --- | --- | --- |
|  | 0.4 M NaCl | 0.6 M KCl | 1.0 M sorbitol | 400 μg/mL Congo Red | 400 μg/mL CFW | 2.5 mM H_2_O_2_ | 5 mM H_2_O_2_ |
| ZN32 | 40.1 ± 2.0c | 57.3 ± 1.4b | 35.4 ± 2.9a | 51.4 ± 1.4a | 34.5 ± 0.7c | 1.6 ± 0.7c | 2.8 ± 0.7c |
| Δ*DaveA* | 60.6 ± 1.2a | 62.1 ± 0.7a | 37.9 ± 2.1a | 43.5 ± 0.7b | 41.6 ± 1.2b | 10.6 ± 1.5a | 18.7 ± 1.9a |
| Δ*DaveA*/*DaVEA* | 41.9 ± 1.2c | 57.3 ± 0.7b | 35.4 ± 1.4a | 51.4 ± 0.7a | 34.5 ± 0.7c | 1.9 ± 0.7c | 2.8 ± 0.7c |
| Δ*DavelB* | 56.8 ± 1.6b | 64.4 ± 2.7a | 35.1 ± 4.5a | 55.2 ± 1.6a | 51.6 ± 2.4a | 7.2 ± 1.1b | 13.6 ± 1.2b |
| Δ*DavelB*/*DaVELB* | 42.8 ± 2.2c | 58.3 ± 1.1b | 36.9 ± 2.9a | 50.8 ± 3.9a | 33.9 ± 2.2c | 1.7 ± 0.7c | 2.8 ± 0.7c |

Tested strains were inoculated on complete medium (CM) supplemented with different stressors and cultured under alternating 12 h/12 h light and dark cycle at 25℃ for 5 days, respectively. These strains were also inoculated on CM medium without any stressors as a control. The final concentrations used were 0.4 M NaCl, 0.6 M KCl, 1.0 M sorbitol, 400 μg/mL Congo Red, 400 μg/mL CFW, 2.5 mM and 5 mM H_2_O_2_. Growth inhibition rate = (diameter of untreated strain – diameter of treated strain)/diameter of untreated strain × 100%. The experiments were repeated three times with the same results. Normality and homogeneity of variance were checked before ANOVA; no data transformation was required. Lowercase represents statistically significant differences among strains (Student–Newman–Keuls test, *P* < 0.05).

**Figure S1.** PCR verification of *D. amygdali* single-gene velvet protein deletion mutants. Genomic DNA of the wild-type strain ZN32 was used as the positive control, and nuclease-free water was used as the negative control.

**Figure S2.** Virulence assay of *D. amygdali* single-gene velvet protein deletion mutants on apple fruits. (a) Apple fruits were surface-sterilized, wounded with a sterile needle, and inoculated with a 5-mm hyphal blocks from the tested strains’ margin. Then, put them at an incubator of 28°C under a 12-h light/12-h dark photoperiod within 90% relative humidity (RH) for 20 days. (b) Lesion diameters were measured at 20 days post-inoculation. (c) Fungal biomass in infected apple tissue was quantified by qRT-PCR. *D. amygdali* histone H3-encoding gene (*DaH3*) was used to detect the fungal content, and the apple translation elongation factor 2 (*TEF2*) was used to normalize different samples. The experiments were repeated three times with the same results. Error bars represent standard deviations of data from three replicates. Lowercase indicates statistically significant differences (Student–Newman–Keuls test, *P* < 0.05).

**Figure S3****.** Deletion of *DaVEA* and *DaVELB* impaired colony surface hydrophobicity in *D. amygdali*. (a) A 10-µL droplet of either 0.02% (w/v) SDS plus 5 mM EDTA solution (detergent treatment, D) or sterile distilled water (control, W) was placed onto the center of each 5-day-old colony, respectively. Droplet collapse was assessed visually after 3 min of incubation at room temperature. (b) Transcript levels of the hydrophobicity-associated genes *DaMHP1* and *DaMPG1* were quantified by qRT-PCR in ZN32 and single-gene velvet protein deletion mutants. The *D. amygdali* histone H3-encoding gene (*DaH3*) served as the endogenous reference gene. The experiments were repeated three times with the same results. Error bars represent standard deviations of data from three replicates. Lowercase indicates statistically significant differences (Student–Newman–Keuls test, *P* < 0.05).

**Figure S4.** Yeast two-hybrid analysis for the interactions among DaVeA, DaVelB, and DaLaeA in *D. amygdali*. Yeast AH109 strain expressing the bait and prey constructs of *DaVEA*, *DaVELB*, and *DaLAEA* were growth on synthetic dextrose medium lacking tryptophan and leucine (SD–Trp/–Leu) for 3 days, followed by photocopying onto SD–Trp/–Leu/–His/–Ade supplemented with 1 mM 3-amino-1,2,4-triazole (3-AT), after incubation at 28°C for another 3 days. Pairs pGBKT7-53/pGADT7 were used as positive control; pGBKT7-Lam/pGADT7 set as negative control. The experiments were repeated three times with the same results.

**Table S1. Primers used in this study.**

|  | **Primer name** | | **Sequence （5’-3’）** | | **Remark** | |
| --- | --- | --- | --- | --- | --- | --- |
|  | *DaVEA*KO-F1 | | TAACTCGAGTGTTTCCTCCCGTCTTGCTCCTT | | Construction of *DaVEA* deletion vector | |
|  | *DaVEA*KO-F2 | | AGGCATTGATGTGTTGACCTCCGTTTGCTGTGGTGGTGCGTG | | Construction of *DaVEA* deletion vector | |
|  | *DaVEA*KO-F3 | | CTCGTCCGAGGGCAAAGGAATAGTAAGGATCCTCTCGTGGCTGTCCTGAACGT | | Construction of *DaVEA* deletion vector | |
|  | *DaVEA*KO-F4 | | TAAGCGGCCGCCGGCGTCGAAGCCGAGGAAAT | | Construction of *DaVEA* deletion vector | |
|  | Sence-*DaVEA* | | ACCTATCAGTGAACCCGAATATCAC | | Construction of *DaVEA* Cas9 vector | |
|  | Anti-*DaVEA* | | AAACGTGATATTCGGGTTCACTGAT | | Construction of *DaVEA* Cas9 vector | |
|  | *DaVEA*-C1 | | ACAGCGCCACCTGCACTGGCCCC | | Verification of *DaVEA* deletion | |
|  | *DaVEA*-C2 | | GTTCTTCACTGGGTCGAAAGCG | | Verification of *DaVEA* deletion | |
|  | *DaVEA*-out | | GACATGTACCTCAGCACCAGTG | | Verification of *DaVEA* deletion | |
|  | *DaVEA*-GFP-F1 | | ACTCACTATAGGGCGAATTGGGTACTCAAATTGGTTATGTACCTCAGCACCAGTGCCTC | | *DaVEA* complementation | |
|  | *DaVEA*-GFP-F2 | | CACCACCCCGGTGAACAGCTCCTCGCCCTTGCTCACGTTCTTCACTGGGTCGAAAGC | | *DaVEA* complementation | |
|  | *DaVELB*KO-F1 | | TGCTCTTCTCAACTCTAAGAGGC | | Construction of *DaVELB* deletion vector | |
|  | *DaVELB*KO-F2 | | AGGCATTGATGTGTTGACCTCCCGTGAGTCCCCGCGGCTGTCGTT | | Construction of *DaVELB*  deletion vector | |
|  | *DaVELB*KO-F3 | | CTCGTCCGAGGGCAAAGGAATAGGGGGCTTCCCGTCTACTACCAC | | Construction of *DaVELB*  deletion vector | |
|  | *DaVELB*KO-F4 | | GCGCGAGCTGGAAGCATGTCTA | | Construction of *DaVELB*  deletion vector | |
|  | Sence-*DaVELB* | | ACCTATCTCGGAACACCGACCATGG | | Construction of *DaVELB*  Cas9 vector | |
|  | Anti-*DaVELB* | | AAACCCATGGTCGGTGTTCCGAGAT | | Construction of *DaVELB* Cas9 vector | |
|  | *DaVELB*-C1 | | CGACTTTGCACCTATGTACCGACC | | Verification of *DaVELB* deletion | |
|  | DaVELB-C2 | | CTCGTCGTCGTCATCATCATC | | Verification of *DaVELB* deletion | |
|  | *DaVELB*-out | | AAGAAGTCACCCTGATGGGCCA | | Verification of *DaVELB* deletion | |
|  | *DaVELB*-GFP-F1 | | ACTCACTATAGGGCGAATTGGGTACTCAAATTGGTTAAGAAGTCACCCTGATGGGCCA | | *DaVELB* complementation | |
|  | *DaVELB*-GFP-F2 | | CACCACCCCGGTGAACAGCTCCTCGCCCTTGCTCACCTCGTCGTCGTCATCATCATC | | *DaVELB* complementation | |
|  | *Da**VELC*KO-F1 | | GTTCTGTACTACTGTAACCACC | | Construction of *DaVELC* deletion vector | |
|  | *DaVELC*KO-F2 | | AGGCATTGATGTGTTGACCTCCGCTACTCTTCGAGTGCTGCCGA | | Construction of *DaVELC* deletion vector | |
|  | *DaVELC*KO-F3 | | CTCGTCCGAGGGCAAAGGAATAGGAACGCTCTGTCCTCTTCTC | | Construction of *DaVELC* deletion vector | |
|  | *DaVELC*KO-F4 | | CCATCTCCGTCCAGATCTTCCA | | Construction of *DaVELC* deletion vector | |
|  | Sence-*DaVELC* | | ACCTACTACTGGAATTACCGAATGG | | Construction of *DaVELC* Cas9 vector | |
|  | Anti-*DaVELC* | | AAACCCATTCGGTAATTCCAGTAGT | | Construction of *DaVELC* Cas9 vector | |
|  | *DaVELC*-C1 | | GAAATCAGCCGCAAACTGAGAC | | Verification of *DaVELC* deletion | |
|  | *DaVELC*-C2 | | CAGGCTGCCGTTCCTTGCTCGCTT | | Verification of *DaVELC* deletion | |
|  | *DaVELC*-out | | ATCCATACACGGCACTTCATCG | | Verification of *DaVELC* deletion | |
|  | *DaVELC*-GFP-F1 | | ACTCACTATAGGGCGAATTGGGTACTCAAATTGGTTATCCATACACGGCACTTCATC | | *DaVELC* complementation | |
|  | *DaVELC*-GFP-F2 | | CACCACCCCGGTGAACAGCTCCTCGCCCTTGCTCACCAGGCTGCCGTTCCTTGCTCGCTT | | *DaVELC* complementation | |
|  | *DaVOSA*KO-F1 | | CAACTGTGGATACTATATGCGC | | Construction of *DaVOSA* deletion vector | |
|  | *DaVOSA*KO-F2 | | AGGCATTGATGTGTTGACCTCCTGCCGCTGCCTGGGTGCGCGCAA | | Construction of *DaVOSA* deletion vector | |
|  | *DaVOSA*KO-F3 | | CTCGTCCGAGGGCAAAGGAATAGTTGGGGCTCTCAGCAATCTGGGT | | Construction of *DaVOSA* deletion vector | |
|  | DaVOSAKO-F4 | | GGCAAGATAGACGAGACGCACA | | Construction of *DaVOSA* deletion vector | |
|  | Sence-*DaVOSA* | | ACCTACACATCTGCAGGTATTACGG | | Construction of *DaVOSA*Cas9 vector | |
|  | Anti-*DaVOSA* | | AAACCCGTAATACCTGCAGATGTGT | | Construction of *DaVOSA* Cas9 vector | |
|  | *DaVOSA*-C1 | | ACATTGCTCCGATGAGTTCCATGC | | Verification of *DaVOSA* deletion | |
|  | *DaVOSA*-C2 | | ATGGCCATCCCTCGGCCCGTAATTG | | Verification of *DaVOSA* deletion | |
|  | *DaVOSA*-out | | ATCGTCTAGAAGCGCACACTCT | | Verification of *DaVOSA* deletion | |
|  | *DaVOSA*-GFP-F1 | | ACTCACTATAGGGCGAATTGGGTACTCAAATTGGTTATCGTCTAGAAGCGCACACTCTC | | *DaVOSA* complementation | |
|  | *DaVOSA*-GFP-F2 | | CACCACCCCGGTGAACAGCTCCTCGCCCTTGCTCACATGGCCATCCCTCGGCCCGTAATT | | *DaVOSA* complementation | |
| RP27-*DaVEA*-F1 | | TTTCGTAGGAACCCAATCTTCAAAATGGTGTCTGTTCTCGGTCCTC | | *DaVEA* complementation | |  |
| RP27-*DaVEA*- F2 | | CACCACCCCGGTGAACAGCTCCTCGCCCTTGCTCACGTTCTTCACTGGGTCGAAAGC | | *DaVEA* complementation | |  |
| RP27-*DaVELB*-F | | TTTCGTAGGAACCCAATCTTCAAAATGGCTTCGTACACTCGAACGC | | *DaVELB* complementation | |  |
| RP27-*DaVELB*-R | | CACCACCCCGGTGAACAGCTCCTCGCCCTTGCTCACCTCGTCGTCGTCATCATCATC | | *DaVELB* complementation | |  |
| RP27- *DaVELC*-F | | TTTCGTAGGAACCCAATCTTCAAAATGACGGTGCGTTCGCTGCTGA | | *DaVELC* complementation | |  |
| RP27-*DaVELC*-R | | CACCACCCCGGTGAACAGCTCCTCGCCCTTGCTCACCAGGCTGCCGTTCCTTGCTCGCTT | | *DaVELC* complementation | |  |
| RP27-*DaVOSA*-F | | TTTCGTAGGAACCCAATCTTCAAAATGGCTGCGTACGACTTTTCAG | | *DaVOSA* complementation | |  |
| RP27-*DaVOSA*-R | | CACCACCCCGGTGAACAGCTCCTCGCCCTTGCTCACATGGCCATCCCTCGGCCCGTAATT | | *DaVOSA* complementation | |  |
| Split-hph-F1 | | GGAGGTCAACACATCAATGCCT | | For gene knockout | |  |
| Split-hph-F2 | | GACGGTGTCGTCCATCACAGTT | | For gene knockout | |  |
| Split-hph-F3 | | ACAGGGTGTCACGTTGCAAGACC | | For gene knockout | |  |
| Split-hph-F4 | | CTATTCCTTTGCCCTCGGACGAG | | For gene knockout | |  |
| RT-*DaMHP1*-F | | AAATGACAAGGATGAACTGGTC | | quantitative RT-PCR analysis | |  |
| RT-*DaMHP1*-R | | ACATGCGAGGTATAGGCCCAGA | | quantitative RT-PCR analysis | |  |
| RT-*DaPTH11*-F | | ACGGTGCAATGACTGATCGGGG | | quantitative RT-PCR analysis | |  |
| RT-*DaPTH11*-R | | CAAACTCGGTGTCCGTCTCACT | | quantitative RT-PCR analysis | |  |
| RT-*ORF1*-F | | AGCATGGTGGCATGACTTTGGA | | quantitative RT-PCR analysis | |  |
| RT-*ORF1*-R | | GACTCGCAGAAGCTCCAGCAA | | quantitative RT-PCR analysis | |  |
| RT-*ORF2*-F | | ACATTTATGTTGGCCCAGAAGA | | quantitative RT-PCR analysis | |  |
| RT-*ORF2*-R | | CTTCTGATAATAGATGTTTGAC | | quantitative RT-PCR analysis | |  |
| RT-*ORF3*-F | | ACCAGTTGCAATTGTGCCAGCC | | quantitative RT-PCR analysis | |  |
| RT-*ORF3*-R | | TTGTCCCTCACGACTTTTGAAG | | quantitative RT-PCR analysis | |  |
| RT-*ORF4*-F | | AGCATACACGGAGCTGTTCG | | quantitative RT-PCR analysis | |  |
| RT-*ORF4*-F | | AGCATACACGGAGCTGTTCG | | quantitative RT-PCR analysis | |  |
| RT-*ORF4*-R | | GACATACGGCTTGACTTGTTCT | | quantitative RT-PCR analysis | |  |
| RT-*ORF5*-F | | TCACCAAAGGCTCGCGTCGGTG | | quantitative RT-PCR analysis | |  |
| RT-*ORF5*-R | | AGCTTCCACCGTAACACGGATA | | quantitative RT-PCR analysis | |  |
| RT-*ORF6*-F | | AAGGATGCGATGGTCTCGAGTC | | quantitative RT-PCR analysis | |  |
| RT-*ORF7*-F | | ACTGAGGACCTTCACCTTAGCA | | quantitative RT-PCR analysis | |  |
| RT-*ORF7*-R | | TCCGACAGAATCCGTGGTTATG | | quantitative RT-PCR analysis | |  |
| RT-*ORF8*-F | | TGTGTATTACATCCGTCAAATC | | quantitative RT-PCR analysis | |  |
| RT-*ORF8*-R | | GGCGTTGAAGACTACTTCAATC | | quantitative RT-PCR analysis | |  |
| RT-*ORF9*-F | | ATCGGTCTTAGGAAGGAGGTG | | quantitative RT-PCR analysis | |  |
| RT-*ORF9*-R | | AACCTCCTTTCTCAAGAGCT | | quantitative RT-PCR analysis | |  |
| RT-*ORF10*-F | | TACGGCAAACATTCCTGTCCTG | | quantitative RT-PCR analysis | |  |
| RT-*ORF10*-R | | ACGATCGATGCTGTCAAAGCTG | | quantitative RT-PCR analysis | |  |
| RT-*ORF11*-F | | ATCTGCTTCTCTGAGGAAGCTG | | quantitative RT-PCR analysis | |  |
| RT-*ORF11*-R | | GTATATCTGGTCTCTAGCCACA | | quantitative RT-PCR analysis | |  |
| RT-*ORF12*-F | | TGATCAGGCGTGGCAAGCAG | | quantitative RT-PCR analysis | |  |
| RT-*ORF12*-R | | AGGCAGCTTTTGCAGCTCCT | | quantitative RT-PCR analysis | |  |
| RT-*ORF13*-F | | AATTACGTGGCCCTGCAGCTGA | | quantitative RT-PCR analysis | |  |
| RT-*ORF13*-R | | TGTGTTTTGTCTCAAAGAGACT | | quantitative RT-PCR analysis | |  |
| QRT-*DaH3*-F | | GAGGTGTCAAGAAGCCTCACC | | quantitative RT-PCR analysis | |  |
| QRT-*DaH3*-R | | GTCCTTCGACTGGATGGTGACA | | quantitative RT-PCR analysis | |  |
| PpTEF2F | | GGTGTGACGATGAAGAGTGATG | | quantitative RT-PCR analysis | |  |
| PpTEF2R | | TGAAGGAGAGGGAAGGTGAAA | | quantitative RT-PCR analysis | |  |
| QRT-13259-F | | AAGCAGCTCCGAGGCTTTGA | | quantitative RT-PCR analysis | |  |
| QRT-13259-R | | AATCTCGTACACTCCCTTCC | | quantitative RT-PCR analysis | |  |
| QRT-9525-F | | GACACTACGGTGTACTCCTT | | quantitative RT-PCR analysis | |  |
| QRT-9525-R | | TCGGACGCTGATGGTGTAGGA | | quantitative RT-PCR analysis | |  |
| QRT-7841-F | | TCAGGCAGCTTCGTGGGTTT | | quantitative RT-PCR analysis | |  |
| QRT-7841-R | | CGAGACGGTCAAAGTGGTCGTT | | quantitative RT-PCR analysis | |  |
| QRT-4968-F | | ACACGACCGTCTACTCGTTC | | quantitative RT-PCR analysis | |  |
| QRT-4968-R | | AGCGACGGTGAGGGAGTAGG | | quantitative RT-PCR analysis | |  |
| QRT-6972-F | | CAGCTACTCATACAGCGACG | | quantitative RT-PCR analysis | |  |
| QRT-6972-R | | GCCCGAAGCGGGCAGCACCCA | | quantitative RT-PCR analysis | |  |
| QRT-8135-F | | GTGTCGACCGCAAGACGGGTA | | quantitative RT-PCR analysis | |  |
| QRT-8135-R | | CAGTCTGTATCCAGCACCTC | | quantitative RT-PCR analysis | |  |
| QRT-7289-F | | GACGAGAATGCCTGGCACTA | | quantitative RT-PCR analysis | |  |
| QRT-7289-R | | CACAGCGTTTCCTCCGGTCA | | quantitative RT-PCR analysis | |  |
| QRT-3468-F | | TGACCATCGAGAACATCTAC | | quantitative RT-PCR analysis | |  |
| QRT-3468-R | | GAGCATTGACTGGCAGGTGA | | quantitative RT-PCR analysis | |  |
| QRT-12691-F | | TGTTCGAGGTCGATCGAACC | | quantitative RT-PCR analysis | |  |
| QRT-12691-R | | AGCCGGGTTATAACCCTTGC | | quantitative RT-PCR analysis | |  |
| QRT-13498-F | | AGAGCTGCACGGCGGGAGGC | | quantitative RT-PCR analysis | |  |
| QRT-13498-R | | CACAAGAACAGGCCATCCTGTC | | quantitative RT-PCR analysis | |  |
| QRT-8135-F | | GTGTCGACCGCAAGACGGGTA | | quantitative RT-PCR analysis | |  |
| QRT-8135-R | | CAGTCTGTATCCAGCACCTC | | quantitative RT-PCR analysis | |  |
| QRT-8306-F | | CATCAATATGACTCACTATTCGT | | quantitative RT-PCR analysis | |  |
| QRT-8306-R | | ATCCAGATATTGCCCCTGAG | | quantitative RT-PCR analysis | |  |
